# Supplementary material for: RNA sequencing-based single sample predictors of molecular subtype and risk of recurrence for clinical assessment of early-stage breast cancer
Source: NPJ Breast Cancer. 2022 Aug 16;8:94. doi: 10.1038/s41523-022-00465-3 (PMC9381586; doi:10.1038/s41523-022-00465-3)
Supplement: Supplementary file 1 — Supplementary Information [file 41523_2022_465_MOESM1_ESM.pdf]

Supplementary Information

RNA Sequencing-Based Single Sample Predictors of Molecular Subtype and Risk of Recurrence for Clinical Assessment of Early-Stage Breast Cancer

Johan Staaf[1,*], Jari Häkkinen[1], Cecilia Hegardt[1], Lao H Saal[1], Siker Kimbung[1], Ingrid Hedenfalk[1], Tonje Lien[2,3], Therese Sørlie[2,4], Bjørn Naume[4,5], Hege Russnes[2,3], Rachel Marcone[6,7], Ayyakkannu Ayyanan[6], Cathrin Brisken[6], Rebecka R. Malterling[8], Bengt Asking[8], Helena Olofsson[9, 10], Henrik Lindman[11], Pär-Ola Bendahl[1], Anna Ehinger[1,12], Christer Larsson[13], Niklas Loman[1,14], Lisa Rydén[15,16], Martin Malmberg[14], Åke Borg[1] and Johan Vallon-Christersson[1,*]

[1]Division of Oncology, Department of Clinical Sciences Lund, Lund University, Medicon Village, SE 22381 Lund, Sweden
[2]Department of Cancer Genetics, Institute for Cancer Research, Oslo University Hospital, POB 4953 Nydalen N-0424, Oslo, Norway
[3]Department of Pathology, Oslo University Hospital, POB 4953 Nydalen N-0424, Oslo, Norway
[4]Institute for Clinical Medicine, Faculty of Medicine, University of Oslo, Oslo, Norway
[5]Department of Oncology, Division of Cancer Medicine, Oslo University Hospital, POB 4953 Nydalen N-0424, Oslo, Norway
[6]ISREC-Swiss Institute for Experimental Cancer Research, School of Life Sciences, Ecole Polytechnique Fédérale de Lausanne, CH-1015 Lausanne, Switzerland
[7]Swiss Institute of Bioinformatics, School of Life Sciences, Ecole Polytechnique Fédérale de Lausanne, CH-1005 Lausanne, Switzerland
[8]Department of Surgery, Region Jönköping County, Jönköping, Sweden
[9]Department of Clinical Pathology, Akademiska hospital, Uppsala, Sweden
[10]Department of Pathology, Centre for Clinical Research of Uppsala University, Vastmanland´s Hospital Västerås, Sweden
[11]Department of Immunology, Genetics and Pathology, Uppsala University Hospital, Uppsala, Sweden
[12]Department of Genetics and Pathology, Laboratory Medicine, Region Skåne, Lund, Sweden
[13]Division of Translational Cancer Research, Department of Laboratory Medicine, Lund University, Lund, Sweden
[14]Department of Hematology, Oncology and Radiation Physics, Skåne University Hospital
[15]Division of Surgery, Department of Clinical Sciences, Lund University, Lund, Sweden
[16]Department of Surgery and Gastroenterology, Skåne University Hospital Malmö

Supplementary Tables 1-5

Supplementary Table 1. Gene-rules included in SSP models for subtypes and ROR.

SSP-PAM50 (5 groups)		SSP-Subtype (4 groups)		SSP-ROR					
Gene rules	Class	Gene rules	Class	Gene rules	Class[1]	cont.	cont.	cont.	cont.
IGF1R<GRB7	Her2	IGF1R<GRB7	Her2	TOP2A<ZNF507	c005	MYBL2<BOC	c035	FAM122A<CCNA2	c065
RERG<CDK12	Her2	RERG<CDK12	Her2	RRM2<MID1	c005	EXO1<CACHD1	c035	TMEM237<MELK	c065
DBNDD2<STARD3	Her2	DBNDD2<STARD3	Her2	UBE2C<TSHZ2	c005	TICRR<GREM2	c035	NEK1<CEP55	c065
SLC39A6<ERBB2	Her2	SLC39A6<ERBB2	Her2	NCAPH<KLHL29	c005	UBE2C<LHFPL6	c035	TTC28<KIF4A	c065
RND2<TCAP	Her2	RND2<TCAP	Her2	BIRC5<CORO2B	c005	BIRC5<MYH11	c035	MAP2K5<CCNB2	c065
ESR1<PGAP3	Her2	ESR1<PGAP3	Her2	MKI67<EVC2	c005	MELK<SPRY2	c035	CDADC1<NCAPG	c065
BCL2<CCNB2	Her2	BCL2<CCNB2	Her2	CCNB2<SNCAIP	c005	BUB1<JAM2	c035	RFX7<BUB1B	c065
PPM1J<C2orf54	Her2	PPM1J<C2orf54	Her2	TPX2<ZNF438	c005	CENPA<C2orf40	c035	PCIF1<UBE2C	c065
CDON<MFSD2A	Her2	CDON<MFSD2A	Her2	KIF4A<EPHB1	c005	CLSPN<SCN4B	c035	ZBTB40<SPAG5	c065
C5AR2<FA2H	Her2	C5AR2<FA2H	Her2	KIF20A<GREM2	c005	CKAP2L<MAMDC2	c035	HMG20A<NUSAP1	c065
RBBP8<RRM2	Her2	TBC1D9<MIEN1	Her2	CCNA2<PDE7B	c005	CCNB2<LAMA2	c035	SNX33<KIFC1	c065
TBC1D9<MIEN1	Her2	RBBP8<RRM2	Her2	PTTG1<ITGA9	c005	CCNA2<MED28	c035	SPEN<PTTG1	c065
STC2<TRIB3	Her2	MAPT<MYBL2	Her2	HMMR<THSD7A	c005	MTFR2<FAT4	c035	WDR33<TPX2	c065
MAPT<MYBL2	Her2	STC2<TRIB3	Her2	DLGAP5<CEP85L	c005	AURKB<NGFR	c035	SIK2<MKI67	c065
IL6ST<ACTR3	Her2	THSD4<CDC20	Her2	NUSAP1<SHE	c005	NCAPH<RHOJ	c035	FBXO42<AURKA	c065
THSD4<CDC20	Her2	IL6ST<ACTR3	Her2	PLK1<PRX	c005	TPX2<ZCCHC24	c035	MNT<PLK1	c065
CCDC170<HJURP	Her2	CCDC170<HJURP	Her2	CKAP2L<NLGN3	c005	MKI67<SLIT2	c035	PTPN21<BUB1	c065
C1orf226<FGFR4	Her2	PCSK6<TMEM86A	Her2	MELK<PRDM5	c005	CEP55<PCDH18	c035	PRMT9<NDC80	c065
PCSK6<TMEM86A	Her2	C1orf226<FGFR4	Her2	AURKA<GPRASP1	c005	HJURP<PAMR1	c035	TRIM65<BIRC5	c065
PARD6B<CENPA	Her2	PARD6B<CENPA	Her2	CDK1<TRIM23	c005	BUB1B<ARHGAP19	c035	RHOJ<KIF20A	c065
TPRG1<SHCBP1	Her2	TPRG1<SHCBP1	Her2	DEPDC1<ZNF891	c005	CDCA5<LMOD1	c035	ZNF429<KIF11	c065
PCM1<GSR	Her2	BUB1<ARHGAP19	LumA	UBE2C<RASL12	c010	SGO1<MAMDC2	c040	MED28<CCNA2	c070
LONRF2<TICRR	Her2	CEP55<MED28	LumA	NUSAP1<FAM122A	c010	TPX2<SH3D19	c040	MAP2K5<CCNB2	c070
NEK10<FUT3	Her2	NCAPG<LCA5	LumA	CEP55<GPRASP1	c010	MYBL2<MYH11	c040	ZNF777<MYBL2	c070
BUB1<ALMS1	LumA	TICRR<TRABD2B	LumA	RRM2<TSHZ2	c010	CEP55<SPRY2	c040	AASS<CKAP2L	c070
CEP55<MED28	LumA	CCNB2<MAP2K5	LumA	TPX2<ST3GAL3	c010	CKAP2L<SCN4B	c040	ZNF691<KIF2C	c070
NCAPG<LCA5	LumA	CCNA2<FAM122A	LumA	CCNA2<C8orf88	c010	CENPA<CLDN11	c040	ASXL1<TPX2	c070
AURKB<PFAS	LumA	AURKB<PFAS	LumA	BUB1<DNAJC18	c010	BUB1<SLIT2	c040	ZER1<UBE2C	c070
CCNB2<MAP2K5	LumA	MYBL2<BOC	LumA	TOP2A<ITGA9	c010	TICRR<TRABD2B	c040	HERC2<MKI67	c070
CCNA2<NEK1	LumA	UBE2C<NCOA5	LumA	DLGAP5<PDE7B	c010	MCM10<TSHZ2	c040	PFAS<AURKB	c070
MYBL2<PPP1R26	LumA	MCM10<GPRASP1	LumA	MELK<PREX2	c010	NCAPH<CEP68	c040	RFXAP<SGO1	c070
RRM2<DIXDC1	LumA	RRM2<DIXDC1	LumA	CCNB2<DCUN1D3	c010	NDC80<ADPRM	c040	ARHGAP19<BUB1	c070
UBE2C<NCOA5	LumA	HJURP<SCN4B	LumA	ANLN<MOB3B	c010	NUF2<ARHGAP19	c040	CTC1<SPAG5	c070
NDC80<ZSCAN30	LumA	NDC80<PRMT9	LumA	ASPM<PDE1C	c010	MELK<LAMA2	c040	PMS1<CEP55	c070
TPX2<CHD6	LumA	TPX2<CHD6	LumA	KIF4A<KLHL29	c010	UBE2C<FBLN5	c040	FAM122A<MELK	c070
KIF4A<UBXN10	LumA	FAM83D<TTC28	LumA	BIRC5<PRDM11	c010	EXO1<PPP1R12B	c040	NMNAT1<KIF4A	c070
SPAG5<STK36	LumA	PLK1<SETD1A	LumA	HJURP<PHYHIP	c010	SHCBP1<CACHD1	c040	ZBTB40<CDCA8	c070
TICRR<TRABD2B	LumA	MELK<RHOJ	LumA	MKI67<PLEKHM3	c010	RACGAP1<DOCK1	c040	DNAJC27<DEPDC1B	c070
PLK1<SETD1A	LumA	MKI67<HERC2	LumA	MYBL2<TESC	c010	KIF20A<LMOD1	c040	WDFY2<CENPA	c070
MKI67<ZBTB40	LumA	PTTG1<CWC27	LumA	NEIL3<TSLP	c010	FAM83D<SIK2	c040	TMEM39B<PLK1	c070
PTTG1<CWC27	LumA	KIF11<IKZF5	LumA	KIF14<PAK3	c010	PLK1<CBX7	c040	CBX7<BIRC5	c070
NUSAP1<MYO5C	LumA	BIRC5<SNX33	LumA	KIF23<ZNF333	c010	BIRC5<TINAGL1	c040	BORCS7<CDK1	c070
HJURP<ZBTB3	LumA	TRIM29<IQGAP3	LumB	CCNB2<SCN4B	c015	RRM2<ZNF106	c045	MRPS14<UBE2T	c075
BIRC5<SNX33	LumA	SFRP1<NEK2	LumB	BUB1<MOB3B	c015	TPX2<SH3D19	c045	CCDC115<UBE2C	c075
KIF11<IKZF5	LumA	KRT5<TPX2	LumB	NDC80<GPRASP1	c015	PRR11<RHOJ	c045	CREBRF<BUB1	c075
MCM10<C4orf36	LumA	MIA<ESPL1	LumB	HJURP<KLHL29	c015	MCM10<SCN4B	c045	TFCP2<TPX2	c075
CDCA5<SLC35E2	LumA	KRT17<UBE2C	LumB	UBE2C<BOC	c015	FAM83D<ARHGEF10	c045	ZYG11B<CEP55	c075
SGO1<CFAP69	LumA	EGFR<HMMR	LumB	MKI67<TSHZ2	c015	CDCA5<SNX33	c045	HERC2<CDCA5	c075
TRIM29<IQGAP3	LumB	KRT16<SPC24	LumB	NUSAP1<RHOJ	c015	SGO1<MSANTD2	c045	TYW3<AURKA	c075
SFRP1<NEK2	LumB	FOXC1<SGO1	LumB	BIRC5<ASB1	c015	BUB1<PARP16	c045	SETD1A<PLK1	c075
KRT5<TPX2	LumB	KLK5<NEIL3	LumB	BUB1B<PLEKHM3	c015	UBE2C<RBFOX2	c045	CEP68<CCNA2	c075
MIA<ESPL1	LumB	KRT6B<CDC25C	LumB	RRM2<ERG	c015	KIFC1<CBX7	c045	MED28<MELK	c075
KRT17<UBE2C	LumB	STAC2<E2F8	LumB	NEK2<CACHD1	c015	TICRR<KLHL3	c045	INTS5<MYBL2	c075
EGFR<HMMR	LumB	KLK6<TICRR	LumB	CEP55<PLA2R1	c015	ANLN<PDGFD	c045	LAMA2<KIF20A	c075
KRT16<SPC24	LumB	DSC3<KIF14	LumB	KIF4A<DNAJC18	c015	KIF4A<ARHGAP19	c045	CALCOCO1<PTTG1	c075
FOXC1<SGO1	LumB	PPP1R14C<POLQ	LumB	TPX2<FBXO42	c015	PLK1<CAMTA2	c045	METTL16<SPAG5	c075
KLK5<NEIL3	LumB	FAM171A1<KIF4A	LumB	TOP2A<SPRY2	c015	NEK2<LZTFL1	c045	PFAS<AURKB	c075
KRT6B<CDC25C	LumB	SERPINB5<CKAP2L	LumB	MELK<C8orf88	c015	RACGAP1<COL4A3BP	c045	ZC3H6<SGO1	c075
STAC2<E2F8	LumB	CRYAB<PTTG1	LumB	PLK1<S1PR2	c015	MELK<CDC37L1	c045	ZBTB40<CDCA8	c075
KLK6<TICRR	LumB	SOX10<KIF18B	LumB	KIF18B<PHYHIP	c015	MKI67<CEP68	c045	TNS2<TACC3	c075
DSC3<KIF14	LumB	ID4<DTL	LumB	ASPM<SAMD5	c015	NUF2<TRIM23	c045	NCOA1<RRM2	c075
PPP1R14C<POLQ	LumB	GABRP<SPC25	LumB	MYBL2<OSR1	c015	HJURP<TTC28	c045	TRABD2B<TICRR	c075
FAM171A1<KIF4A	LumB	KRT14<UBE2T	LumB	CCNA2<MAMDC2	c015	EXO1<NAPEPLD	c045	ARHGAP19<NDC80	c075
SERPINB5<CKAP2L	LumB	FOXA1<ATP2C1	Basal	KIF20A<TSHZ2	c020	BOC<UBE2C	c050	MTMR10<CEP55	c080
SOX10<KIF18B	LumB	MLPH<SLC43A3	Basal	CEP55<CACHD1	c020	TSHZ2<BUB1	c050	CEP68<BUB1	c080
CRYAB<PTTG1	LumB	PRR15<FOXC1	Basal	BIRC5<TTC28	c020	CACHD1<NCAPH	c050	EZH1<SPAG5	c080
ID4<DTL	LumB	TTC6<BCL11A	Basal	MELK<LCA5	c020	STAC<KIF14	c050	GID4<PRR11	c080
GABRP<SPC25	LumB	AGR2<RARRES1	Basal	MKI67<CLDN11	c020	SMAD9<ORC6	c050	ZBTB20<HJURP	c080
KRT14<UBE2T	LumB	TBC1D9<RBMS1	Basal	TPX2<CEP68	c020	CLDN11<CCNB2	c050	ACTR8<AURKA	c080
BOC<ASF1B	LumB	ZMYND10<TMCC2	Basal	KIF2C<SCN4B	c020	CYYR1<RRM2	c050	TNS2<MYBL2	c080
DSG3<E2F7	LumB	ATP8B1<DSC2	Basal	UBE2C<CBX7	c020	CEP55<SOCS5	c050	CCNDBP1<RRM2	c080
SYNM<PBK	LumB	AGR3<GABRP	Basal	DEPDC1<SAMD5	c020	SAMD5<SKA1	c050	POFUT1<TPX2	c080
MLPH<SLC43A3	Basal	THSD4<MAML2	Basal	MYBL2<IL34	c020	SLIT2<TPX2	c050	PKIG<UBE2C	c080
FOXA1<PLSCR1	Basal	SPDEF<TSC22D4	Basal	CCNB2<EGFR	c020	SCN3B<POLQ	c050	ATG14<DLGAP5	c080
PRR15<FOXC1	Basal	CERS6<ANP32E	Basal	RRM2<SPRY2	c020	PSAT1<ERCC6L	c050	SLU7<PTTG1	c080
TTC6<TBX19	Basal	SLC44A4<KLF5	Basal	HJURP<GPRASP1	c020	C8orf88<EXO1	c050	DCAKD<BIRC5	c080
AGR2<RARRES1	Basal	ANXA9<CDCA7	Basal	BUB1<MAMDC2	c020	OSR1<MYBL2	c050	ZNF592<CDCA5	c080
ATP8B1<DSC2	Basal	ZNF552<SFT2D2	Basal	EXO1<GNAL	c020	ALX4<MCM10	c050	CAMTA2<PLK1	c080
TSPAN1<PSAT1	Basal	C9orf152<PSAT1	Basal	CDK1<SOCS5	c020	FOXC1<NDC80	c050	TIMM22<AURKB	c080
TBC1D9<UBE2J1	Basal	ESR1<FAM171A1	Basal	KIF4A<WDFY2	c020	TMOD1<HJURP	c050	LRIG2<CKAP2L	c080
BCAS1<BCL11A	Basal	GATA3<RANBP1	Basal	MCM10<LRP4	c020	MOB3B<SPC25	c050	NAF1<CCNA2	c080
AGR3<GPM6B	Basal	XBP1<YBX1	Basal	NCAPH<ITGA9	c020	SCN4B<KIF20A	c050	WDR19<KIF4A	c080
ZMYND10<TMCC2	Basal	CAPN8<PPP1R14C	Basal	KIF11<RFX7	c020	RGMA<PLK1	c050	ARNT<UBE2T	c080
SLC44A4<KLF5	Basal	DNAH5<CHODL	Basal	FOXM1<MAPKBP1	c020	MATN2<PTTG1	c050	CDC37L1<MELK	c080
ANXA9<CDCA7	Basal			MELK<CACHD1	c025	SCN4B<KIF4A	c055	SIK3<SPAG5	c085
SPDEF<TSC22D4	Basal			PLK1<NGFR	c025	C8orf88<HJURP	c055	TOPORS<MELK	c085
TFF3<KCNN4	Basal			CCNB2<SPRY2	c025	BOC<CDC20	c055	ZNF280D<CEP55	c085
CERS6<ANP32E	Basal			KIF4A<TSHZ2	c025	IL11RA<PLK1	c055	KDM3B<TPX2	c085
GATA3<MRPL9	Basal			CDC20<BOC	c025	LRCH1<TPX2	c055	BICRAL<CCNA2	c085
ZNF552<SFT2D2	Basal			CEP55<TMEM237	c025	NIPSNAP3B<MCM10	c055	ERCC6<KIF4A	c085
ESR1<NCAPH	Basal			MKI67<ITGA9	c025	SYN2<TICRR	c055	ZNF621<CENPA	c085
C9orf116<CCNE1	Basal			KIF15<GPRASP1	c025	ITGA9<MKI67	c055	TGFBRAP1<NCAPH	c085
CAPN8<PPP1R14C	Basal			BUB1<GPRASP2	c025	SLIT2<RRM2	c055	CCDC71<MYBL2	c085
THSD4<PTPN14	Basal			TPX2<AMOTL1	c025	EGFR<BUB1	c055	CRIM1<UBE2T	c085
DEGS2<KCNK5	Basal			CENPA<HLF	c025	JAM2<CCNB2	c055	ZC3H6<CKAP2L	c085
DNAH5<CHODL	Basal			UBE2C<MYH11	c025	SMAD9<DEPDC1B	c055	IL17RD<SGO1	c085
CGN<COL17A1	Normal			EXO1<SCN4B	c025	NGFR<MYBL2	c055	ZBTB20<MCM10	c085
MYB<MYH11	Normal			KIF14<SAMD5	c025	TP63<EXO1	c055	LRP6<FAM83D	c085
RSPH3<TSHZ2	Normal			POLQ<THSD7B	c025	TCEAL7<NCAPG	c055	SNX15<CDCA5	c085
OPA1<PROS1	Normal			HJURP<HOXA5	c025	GPRASP1<SHCBP1	c055	TNKS<BUB1	c085
C2orf15<KLHL13	Normal			KIF18B<TRABD2B	c025	PREX2<CENPE	c055	VAMP2<UBE2C	c085
COG7<MATN2	Normal			MCM10<KLHL29	c025	MMRN2<UBE2C	c055	RBM22<PTTG1	c085
SPATA17<PDE1C	Normal			DLGAP5<DMD	c025	TSHZ2<KIF2C	c055	LCA5<MTFR2	c085
TESMIN<C2orf40	Normal			CCNA2<CLDN11	c025	FOXC1<CDC45	c055	MCC<CDC45	c085
OVOL2<NGFR	Normal			RRM2<LAMA2	c025	CEP68<PRC1	c055	SORBS1<ANLN	c085
CIPC<SPRY2	Normal			HJURP<SCN4B	c030	C20orf194<CEP55	c060	STX12<RRM2	c090
TRPS1<SFRP1	Normal			MCM10<GPRASP1	c030	GPRASP1<MCM10	c060	TMEM35B<CCNB2	c090
BLZF1<CFI	Normal			KIF4A<CLDN11	c030	LAMA2<TPX2	c060	MARCH8<NCAPH	c090
ESR1<KRT5	Normal			CENPA<MAMDC2	c030	ZCCHC24<UBE2C	c060	TMEM222<MYBL2	c090
MYO5B<SYNM	Normal			MYBL2<COL17A1	c030	SHE<HJURP	c060	TNS2<CDCA5	c090
MYO6<DST	Normal			BUB1<C20orf194	c030	SCN4B<CDC45	c060	SMAD4<CEP55	c090
CLUAP1<IL33	Normal			MELK<CACHD1	c030	MEIS2<KIF4A	c060	CRIM1<RACGAP1	c090
CCDC170<SCN4B	Normal			NCAPG<LCA5	c030	RNF180<CENPE	c060	MINDY1<BIRC5	c090
SMIM22<KRT14	Normal			UBE2C<MYH11	c030	CCDC8<MYBL2	c060	ZBTB20<CKAP2L	c090
ZMYND10<MIA	Normal			AURKB<RASL12	c030	SPRY2<CCNB2	c060	SIK3<BUB1	c090
GLYR1<PLPP3	Normal			PRR11<TSHZ2	c030	ARHGAP23<CDCA5	c060	CX3CR1<MTFR2	c090
B4GALNT4<OSR1	Normal			CEP55<RHOJ	c030	PGM5<BUB1	c060	UBIAD1<KIF2C	c090
SIAH2<CCL14	Normal			TPX2<POLR1E	c030	TCEANC2<CKAP2L	c060	YIPF4<CENPA	c090
CELSR1<TINAGL1	Normal			RRM2<PROS1	c030	MAPKBP1<SPAG5	c060	PLSCR4<MELK	c090
PLEKHA3<C8orf88	Normal			CCNB2<AMOTL1	c030	TCEAL7<SGO1	c060	POMGNT2<E2F1	c090
				CCNA2<IL17RD	c030	CACHD1<MELK	c060	KIF3B<TPX2	c090
				PLK1<BOC	c030	SIRT1<CDK1	c060	KLHL21<AURKB	c090
				SGO1<TP63	c030	FAM13C<CENPA	c060	CLCN6<SGO1	c090
				BIRC5<LMOD1	c030	ZBTB20<MKI67	c060	CBX7<TROAP	c090
				NDC80<PPP1R12B	c030	TTBK2<BUB1B	c060	ZNF76<KIFC1	c090
				EXO1<C2orf40	c030	FBXO34<TOP2A	c060	DOCK1<MKI67	c090

[1]Classes for SSP-ROR correspond to ROR ranges: c005 (<5), c010 (6-10), c015 (11-15), c020 (16-20), c025 (21-25), c030 (26-30), c035 (31-35), c040 (36-40), c045 (41-45), c050 (46-50), c055 (51-55), c060 (56-60), c065 (61-65), c070 (66-70), c075 (71-75), c080 (76-80), c085 (81-85), c090 (86-90).

Supplementary Table 2. Gene-rules included in SSP models for clinical markers.

SSP-ER		SSP-PR		SSP-HER2		SSP-HER2 (ER+)		SSP-HER2 (ER-)		SSP-Ki67		SSP-NHG	
**Gene rules**	**Class**	**Gene rules**	**Class**	**Gene rules**	**Class**	**Gene rules**	**Class[1]**	**Gene rules**	**Class[2]**	**Gene rules**	**Class**	**Gene rules**	**Class**
LPIN1<ESR1	Positive	THAP2<PGR	Positive	ERBB2<ATP1A1	Negative	STARD3<BET1L	HER2nERp	ERBB2<PSMD8	HER2nERn	MYBL2<GPN2	Low	MYBL2<RILPL2	Grade 1
DISC1<CCDC170	Positive	LAD1<MAPT	Positive	MIEN1<SARS	Negative	GRB7<ZNF395	HER2nERp	MIEN1<TMEM147	HER2nERn	MKI67<ERCC6	Low	UBE2C<SIK3	Grade 1
PADI2<AGR3	Positive	GMIP<FGD3	Positive	PGAP3<WDTC1	Negative	MIEN1<CLSTN1	HER2nERp	PGAP3<DHX16	HER2nERn	TPX2<UTP14C	Low	BIRC5<PTPN21	Grade 1
ATP2C1<TBC1D9	Positive	PGR<THAP2	Negative	STARD3<CIZ1	Negative	PGAP3<WDTC1	HER2nERp	GRB7<RFXANK	HER2nERn	UBE2C<ALAD	Low	MYBL2<ZFYVE1	Grade 2
PSAT1<GREB1	Positive	MAPT<LAD1	Negative	GRB7<EHMT2	Negative	ERBB2<CD151	HER2nERp	STARD3<BABAM1	HER2nERn	PLK1<RIOX1	Low	CCNA2<METTL14	Grade 2
KHSRP<GATA3	Positive	FGD3<GMIP	Negative	TCAP<PSPN	Negative	GSDMB<CFAP69	HER2nERp	TCAP<KCNJ14	HER2nERn	PTTG1<CWC27	Low	AURKB<CTC1	Grade 2
INAVA<ANXA9	Positive			PSMD3<RAB13	Negative	MED1<PRKRA	HER2nERp	ORMDL3<ATP5L	HER2nERn	MELK<RAB33B	Low	ZFYVE1<MYBL2	Grade 3
GGCX<THSD4	Positive			CDK12<ASH1L	Negative	PSMD3<MRPL20	HER2nERp	PSMD8<ERBB2	HER2pERn	CCNB2<ZBTB44	Low	CIPC<TPX2	Grade 3
PPP1R14C<SERPINA11	Positive			ATP1A1<ERBB2	Positive	BET1L<STARD3	HER2pERp	TMEM147<MIEN1	HER2pERn	KIF4A<ZNF619	Low	APH1B<AURKA	Grade 3
PFKP<CA12	Positive			SARS<MIEN1	Positive	ZNF395<GRB7	HER2pERp	DHX16<PGAP3	HER2pERn	CDCA5<TMEM242	Low		
BEND3<C5AR2	Positive			WDTC1<PGAP3	Positive	CLSTN1<MIEN1	HER2pERp	RFXANK<GRB7	HER2pERn	CDCA8<FAM161B	Low		
FUT3<TTC6	Positive			CIZ1<STARD3	Positive	WDTC1<PGAP3	HER2pERp	BABAM1<STARD3	HER2pERn	MCM10<ZNF214	Low		
HAPLN3<SUSD3	Positive			EHMT2<GRB7	Positive	CD151<ERBB2	HER2pERp	KCNJ14<TCAP	HER2pERn	GPN2<MYBL2	High		
SLC15A2<ERBB4	Positive			PSPN<TCAP	Positive	CFAP69<GSDMB	HER2pERp	ATP5L<ORMDL3	HER2pERn	ERCC6<MKI67	High		
SRPK1<ARMT1	Positive			RAB13<PSMD3	Positive	PRKRA<MED1	HER2pERp			UTP14C<TPX2	High		
DSC2<NAT1	Positive			ASH1L<CDK12	Positive	MRPL20<PSMD3	HER2pERp			ALAD<UBE2C	High		
CCT8<SLC39A6	Positive									RIOX1<PLK1	High		
CDK6<CLSTN2	Positive									CWC27<PTTG1	High		
NUDT8<DEGS2	Positive									RAB33B<MELK	High		
ESR1<LPIN1	Negative									ZBTB44<CCNB2	High		
CCDC170<DISC1	Negative									ZNF619<KIF4A	High		
AGR3<PADI2	Negative									TMEM242<CDCA5	High		
TBC1D9<ATP2C1	Negative									FAM161B<CDCA8	High		
GREB1<PSAT1	Negative									ZNF214<MCM10	High		
GATA3<KHSRP	Negative												
ANXA9<INAVA	Negative												
THSD4<GGCX	Negative												
SERPINA11<PPP1R14C	Negative												
CA12<PFKP	Negative												
C5AR2<BEND3	Negative												
TTC6<FUT3	Negative												
SUSD3<HAPLN3	Negative												
ERBB4<SLC15A2	Negative												
ARMT1<SRPK1	Negative												
NAT1<DSC2	Negative												
SLC39A6<CCT8	Negative												
CLSTN2<CDK6	Negative												
DEGS2<NUDT8	Negative												

[1]Classes for SSP-HER2 (specific for ER+): HER2nERp (Negative), HER2pERp (Positive). [2]Classes for SSP-HER2 (specific for ER-): HER2nERn (Negative), HER2pERn (Positive).

Supplementary Table 3. SSP performance validated against clinicopathological re-stain and re-assessed consensus data in the independent ABiM material from Brueffer et al.[1]

SSP model vs. pathology	Accuracy	Accuracy Null	Kappa	Class value	Sensitivity	Specificity	Positive predictive value	Negative predictive value
ER status, n=405	0.97 (0.94, 0.98)	0.78	0.90 (0.85, 0.95)	Positive	0.96	0.98	0.99	0.88
PR status, n=405	0.84 (0.80, 0.87)	0.66	0.67 (0.59, 0.74)	Positive	0.81	0.91	0.94	0.71
HER2 status, n=405	0.80 (0.76, 0.84)	0.79	0.55 (0.47, 0.63)	Positive	0.95	0.76	0.52	0.98
HER2 status[2], n=405	0.88 (0.85, 0.91)	0.79	0.69 (0.61, 0.77)	Positive	0.93	0.87	0.66	0.98
Ki67 status, n=405	0.67 (0.63, 0.72)	0.57	0.39 (0.32, 0.46)	High	0.98	0.44	0.57	0.97
NHG, n=405	0.65 (0.60, 0.69)	0.47	0.41 (0.34, 0.48)	Grade 1	0.60	0.89	0.43	0.94
				Grade 2	0.32	0.92	0.73	0.66
				Grade 3	0.95	0.60	0.68	0.93

Figures in parentheses represent 95% confidence interval. [1]Brueffer, C., et al. *JCO Precis Oncol* **2**(2018). [2]HER2 status by SSP model for ER specific HER2 status.

Supplementary Table 4. SSP model *vs.* Prosigna model when evaluating identically sourced RNA and Prosigna model evaluation using fresh frozen tissue (FT) *vs.* FFPE material in the independent external clinical series ABiM.

Comparison and models	Accuracy	Accuracy Null	Kappa	Class value	Sensitivity	Specificity	Positive predictive value	Negative predictive value
SSP vs. Prosigna[1] FT								
Subtype, n=100	0.87 (0.79, 0.93)	0.40	0.81 (0.72, 0.91)	Luminal A	0.91	0.94	0.89	0.95
				Luminal B	0.83	0.98	0.97	0.89
				Basal	0.81	1.00	1.00	0.97
				Her2-enriched	1.00	0.91	0.53	1.00
ROR risk classification, n=98	0.79 (0.69, 0.86)	0.58	0.61 (0.48, 0.74)	Low	0.73	0.90	0.58	0.95
				Intermediate	0.42	0.94	0.73	0.82
				High	0.96	0.78	0.86	0.94
Binary ROR risk categorization, n=98	0.89 (0.81, 0.94)	0.58	0.76 (0.63, 0.89)	Low/Intermediate	0.78	0.96	0.94	0.86
				High	0.96	0.78	0.86	0.94
Prosigna[2] FT vs. FFPE								
Subtype, n=100	0.82 (0.73, 0.89)	0.38	0.74 (0.63, 0.85)	Luminal A	0.79	0.92	0.86	0.88
				Luminal B	0.85	0.83	0.73	0.92
				Basal	1.00	0.98	0.88	1.00
				Her2-enriched	0.64	1.00	1.00	0.95
ROR risk classification, n=98	0.84 (0.75, 0.90)	0.60	0.71 (0.59, 0.84)	Low	0.70	0.99	0.93	0.93
				Intermediate	0.79	0.86	0.58	0.94
				High	0.90	0.90	0.93	0.85
Binary ROR risk categorization, n=98	0.90 (0.82, 0.95)	0.60	0.79 (0.67, 0.91)	Low/Intermediate	0.90	0.90	0.85	0.93
				High	0.90	0.90	0.93	0.85

Figures in parentheses represent 95% confidence interval. [1]Prosigna reference is Nanostring data (not clinical test) generated in Lund on fresh tissue (FT) and sent to Nanostring for calculating the Prosigna Subtype and ROR readout (performance evaluated in ABiM, all available cases). [2]Prosigna is Nanostring data (not clinical test) generated in Lund on fresh tissue (FT) and on FFPE material as prescribed and sent to Nanostring for calculating the Prosigna Subtype and ROR readout (performance evaluated in ABiM, all available cases).

Supplementary Table 5. Concordance analysis of emulated treatment recommendation between SSP and Prosigna for ER+ HER2- N0 cases in the external clinical series OSLO2-EMIT0 and ABiM.

Comparison and series	Accuracy	Accuracy Null	Kappa	Class value	Sensitivity	Specificity	Positive predictive value	Negative predictive value
SSP vs. Prosigna[1] in OSLO2-EMIT0, n=54								
ETR	0.76 (0.62, 0.87)	0.54	0.61 (0.43, 0.80)	None	0.67	0.95	0.80	0.91
				Endo	0.72	0.80	0.81	0.71
				ChemoEndo	0.92	0.85	0.67	0.97
ETR dichotomized for chemotherapy	0.87 (0.75, 0.95)	0.76	0.69 (0.48, 0.90)	None/Endo	0.85	0.92	0.97	0.67
				ChemoEndo	0.92	0.85	0.67	0.97
SSP vs. Prosigna[2] in ABiM, n=33								
ETR	0.82 (0.65, 0.93)	0.45	0.70 (0.48, 0.93)	None	1.00	1.00	1.00	1.00
				Endo	0.80	0.83	0.80	0.83
				ChemoEndo	0.77	0.85	0.77	0.85
ETR dichotomized for chemotherapy	0.82 (0.65, 0.93)	0.61	0.62 (0.34, 0.89)	None/Endo	0.85	0.77	0.85	0.77
				ChemoEndo	0.77	0.85	0.77	0.85
SSP vs. Prosigna[3] in pooled OSLO2-EMIT0 and ABiM, n=87								
ETR	0.78 (0.68, 0.86)	0.51	0.65 (0.51, 0.79)	None	0.76	0.97	0.87	0.94
				Endo	0.75	0.81	0.80	0.76
				ChemoEndo	0.85	0.85	0.71	0.93
ETR dichotomized for chemotherapy	0.85 (0.77, 0.92)	0.70	0.66 (0.50, 0.83)	None/Endo	0.85	0.85	0.93	0.71
				ChemoEndo	0.85	0.85	0.71	0.93

Figures in parentheses represent 95% confidence interval. ETR: emulated treatment recommendation. [1]Prosigna reference in OSLO2-EMIT0 is the clinical test performed on FFPE as prescribed. [2]Prosigna reference in ABiM is Nanostring data (not clinical test) generated in Lund on FFPE material as prescribed and sent to Nanostring for calculating the Prosigna Subtype and ROR readout. [3]Prosigna results for OSLO2-EMIT0 cases are from the clinical test performed on FFPE as prescribed. Prosigna results for ABiM cases are from Nanostring data (not clinical test) generated in Lund on FFPE material as prescribed and sent to Nanostring for calculating the Prosigna Subtype and ROR readout.

Supplementary Figures 1-6

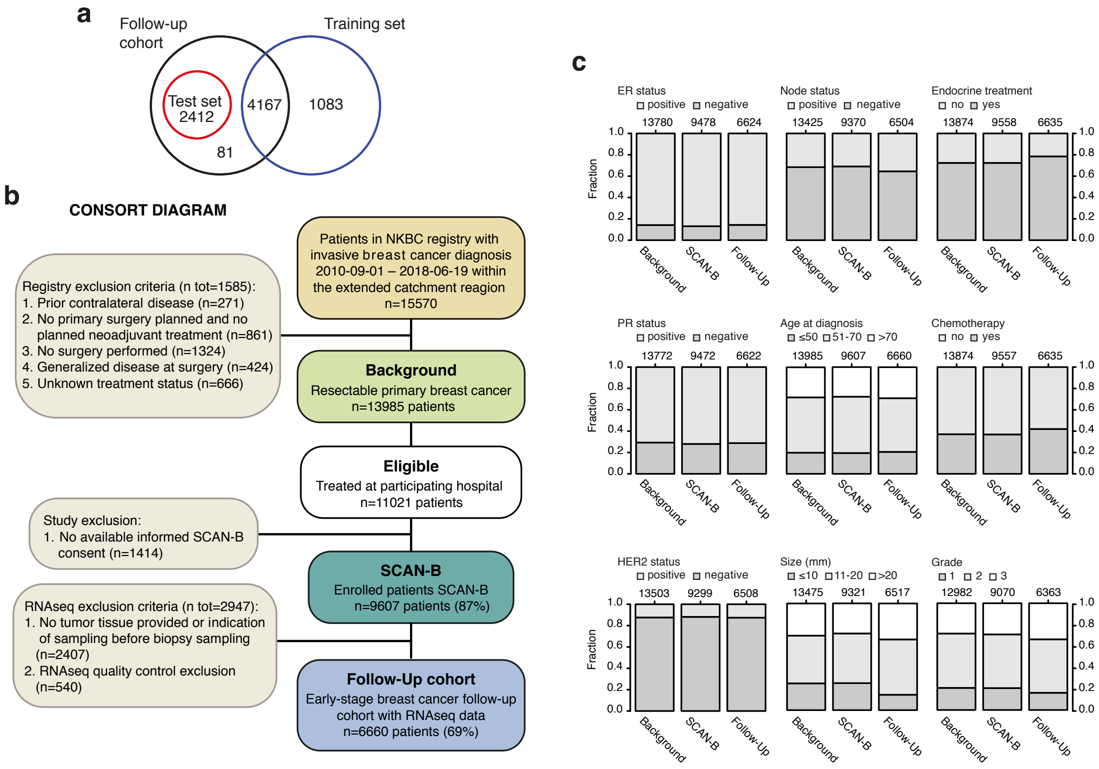

Supplementary Figure 1. Venn diagram of patient overlap in the SCAN-B study material cohorts and consort diagram of patient selection for the early-stage follow-up cohort with bar charts illustrating population-based representativeness compared to the background population. a, Venn diagram of patient overlap for the follow-up cohort of early breast cancer (black), the training set (blue), and independent test set (red). **b**, Consort diagram of patient inclusion in the follow-up cohort of early breast cancer. **c**, Bar charts of incidence for important clinicopathological variables in the background population (left), enrolled SCAN-B patients (center), and SCAN-B patients with RNA-sequencing data (right) in the follow-up cohort. NKBC: Swedish National Quality Register for Breast Cancer.

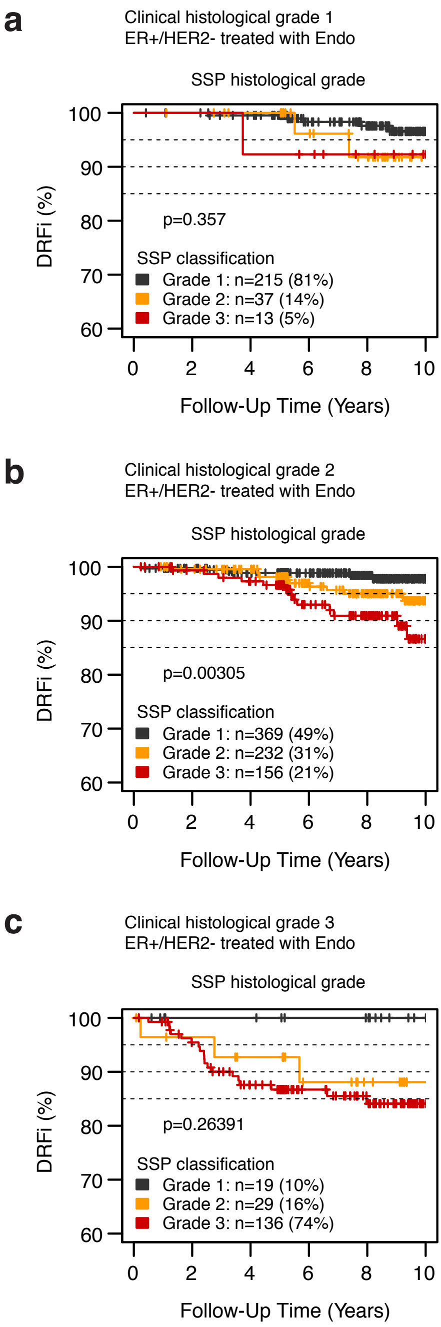

Supplementary Figure 2. Kaplan-Meier plots for SSP classification of NHG. Plots are for groups of separate clinical NHG grade in patients diagnosed with ER+/HER2- breast cancer and that only received endocrine adjuvant treatment: **a**, clinical NHG 1, **b**, clinical NHG 2, **c**, clinical NHG 3.

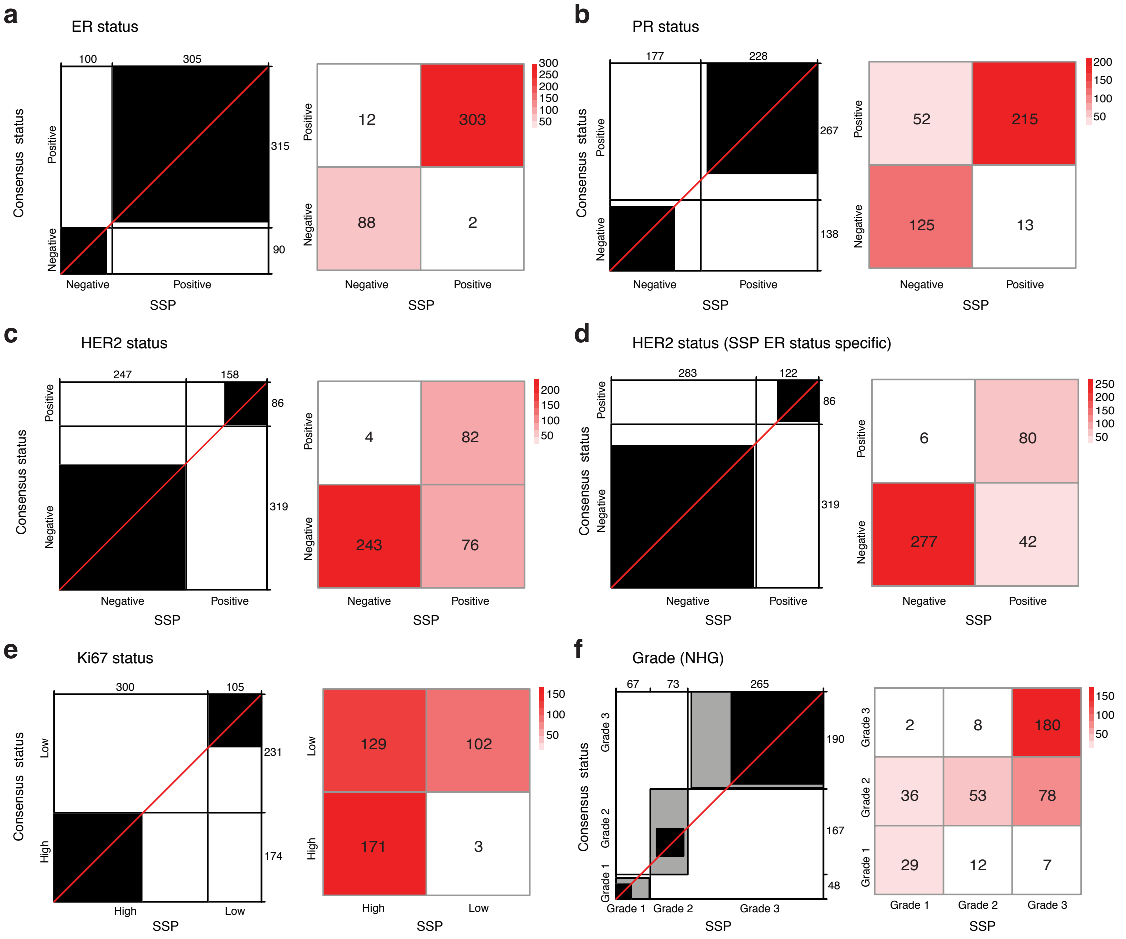

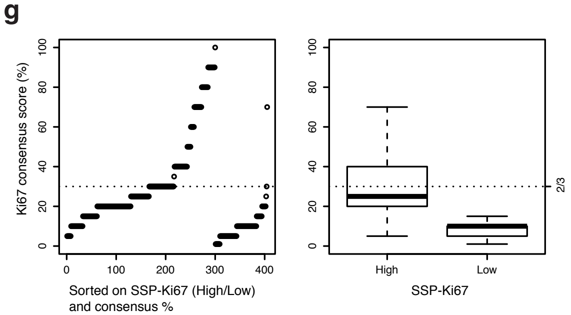

Supplementary Figure 3. Validation of SSP models for clinical markers against consensus status in the external clinical ABiM series. Agreement chart and confusion matrix comparing the SSP classifications (x-axis/columns) with consensus histopathology status (y-axis/rows) for **a**, ER status, **b**, PR status, **c**, HER2 status using a general SSP model, **d**, HER2 status using a SSP model specific for ER status, **e**, Ki67-status, **f**, NHG, **g**, Ki67 consensus scoring (%) by SSP Ki67 classification: samples plotted by SSP classification (left plot) and box-plots (right plot), horizontal dotted line is the two thirds (2/3) percentile. Boxplot elements: center line (median), bounds of box (upper and lower quartiles), whiskers (1.5x interquartile range).

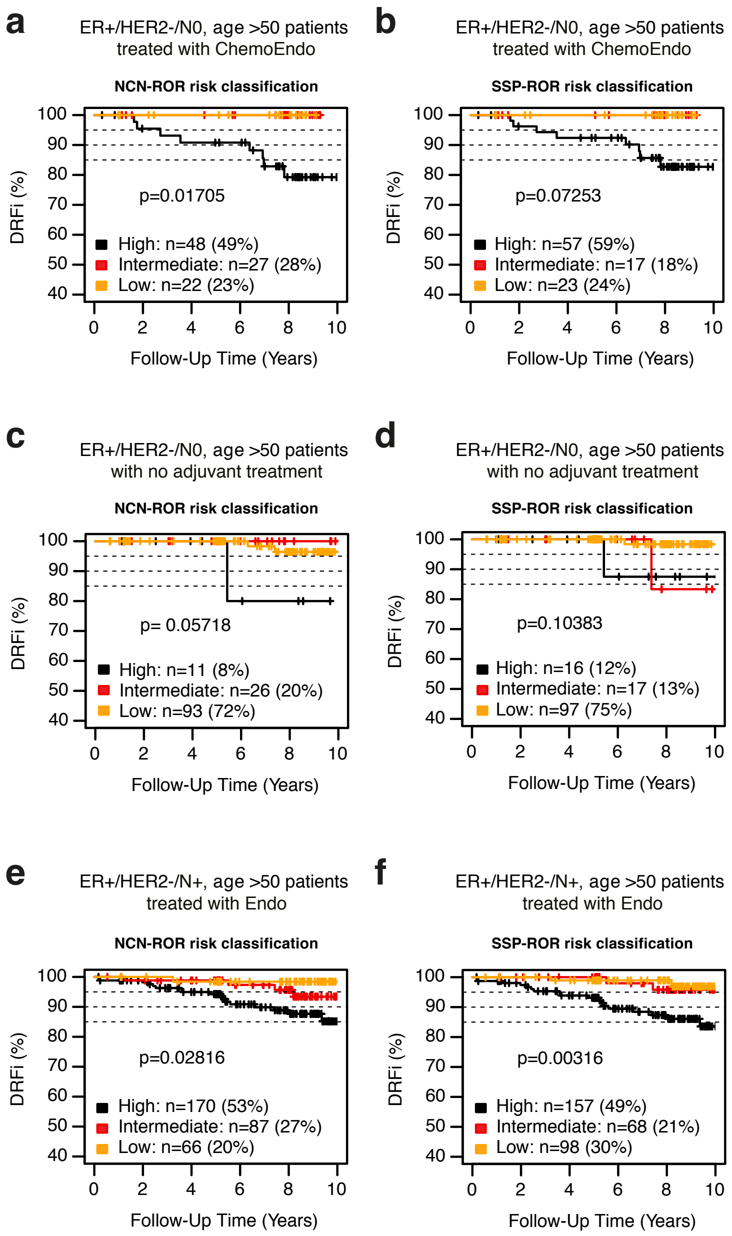

Supplementary Figure 4. Comparing SSP and NCN classifications in the independent population-based test set by assessment of prognostic value. a, Kaplan-Meier plots for stratification by ROR risk classification from SSP-ROR risk category and **b**, NCN-ROR risk category in ER+/HER2-/N0 disease diagnosed over 50 years of age treated with chemotherapy in addition to endocrine adjuvant treatment. **c**, Kaplan-Meier plots for stratification by ROR risk classification from SSP-ROR risk category and **d**, NCN-ROR risk category in ER+/HER2-/N0 disease diagnosed over 50 years of age that did not receive any adjuvant treatment. **e**, Kaplan-Meier plots for stratification by ROR risk classification from SSP-ROR risk category and **f**, NCN-ROR risk category in ER+/HER2-/N+ disease diagnosed over 50 years of age treated with endocrine adjuvant treatment alone.

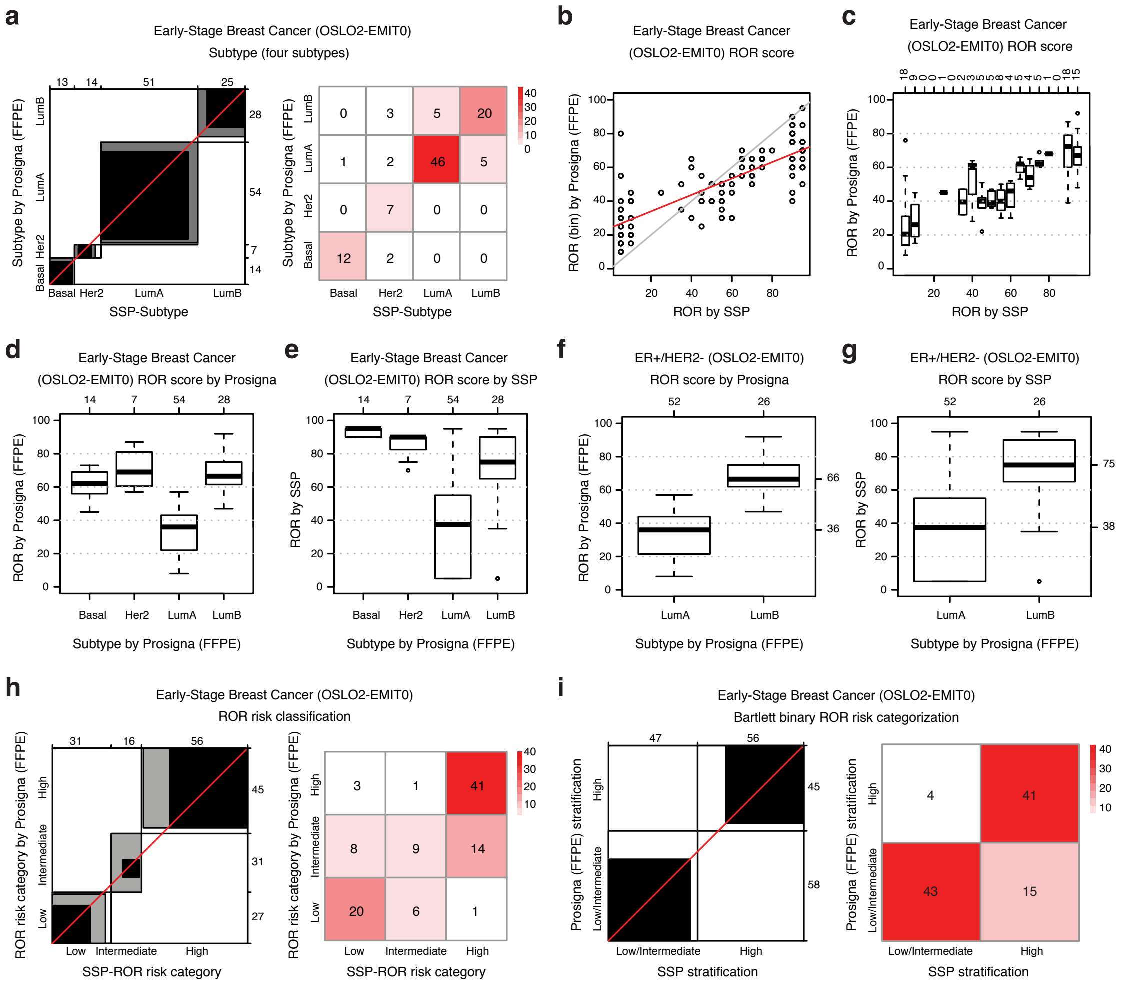

Supplementary Figure 5. Comparing SSP classifications from fresh frozen tissue against the clinical Prosigna test from FFPE tissue in an independent clinical series of primary breast cancer (OSLO2-EMIT0). a, Agreement chart and confusion matrix comparing SSP classifications (x-axis/columns) with Prosigna classifications (y-axis/rows) for Subtype (four subtypes). **b**, Scatterplot of binned Prosigna ROR values (y-axis) versus SSP-ROR (x-axis). **c**, Boxplots of Prosigna ROR values (y-axis) by SSP-ROR (x-axis). **d**, Distributions of Prosigna ROR values and **e**, SSP-ROR values by Prosigna classification for Subtype (four subtypes). **f**, Distributions of Prosigna ROR values and **g**, SSP-ROR values by Prosigna classification for Subtype (four subtypes) for ER+/HER2- breast cancer classified as Luminal A or Luminal B. **h**, Agreement chart and confusion matrix comparing SSP classification (x-axis/columns) with Prosigna classification (y-axis/rows) for ROR risk category and **i**, the two-group ROR stratification according to Bartlett et al. [5]. Boxplot elements: center line (median), bounds of box (upper and lower quartiles), whiskers (1.5x interquartile range).

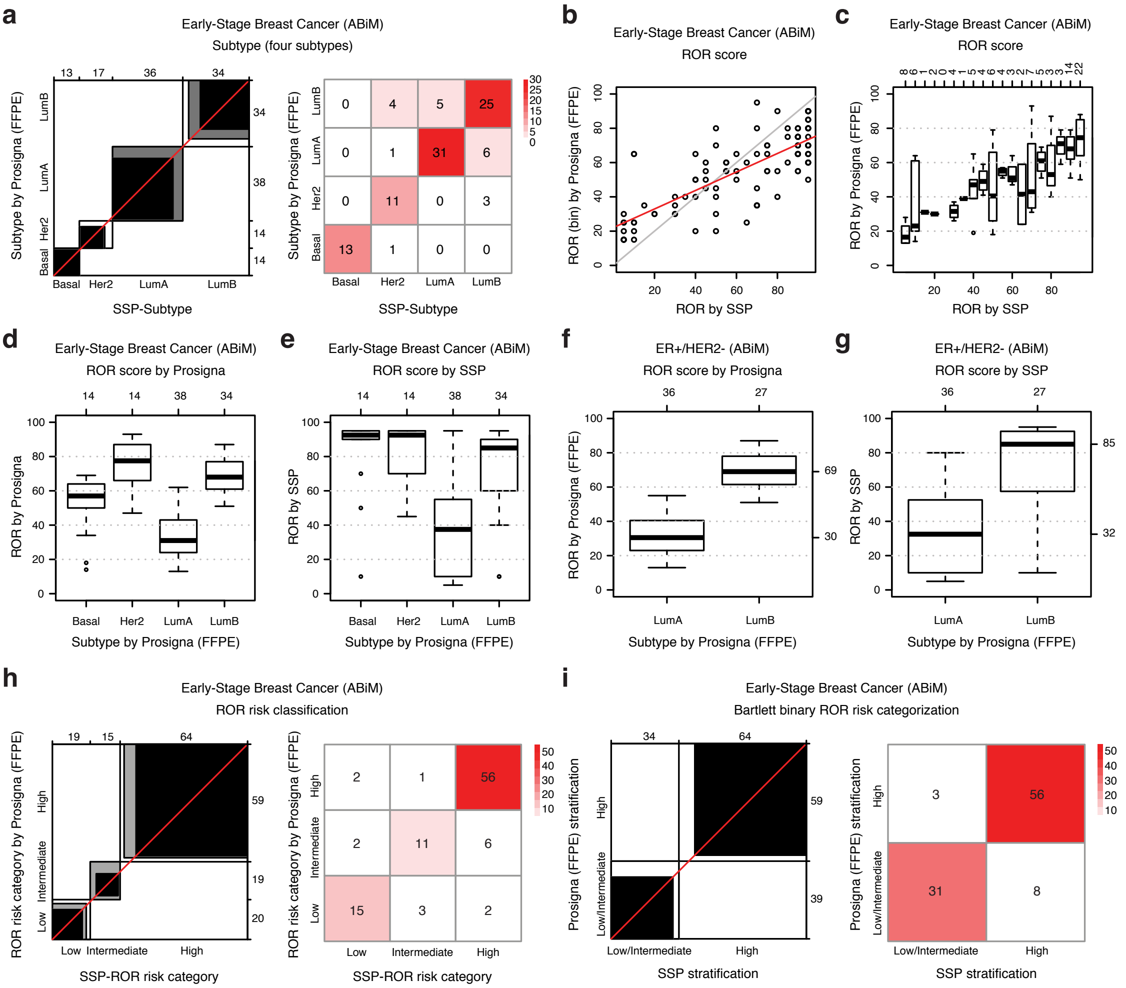

Supplementary Figure 6. Comparing SSP classifications from fresh frozen tissue against the Prosigna model on Nanostring data from FFPE tissue in an independent clinical series of early breast cancer (ABiM). a, Agreement chart and confusion matrix comparing SSP classifications (x-axis/columns) with matched Prosigna classifications (y-axis/rows) for Subtype (four subtypes). **b**, Scatterplot of binned Prosigna ROR values (y-axis) versus SSP-ROR (x-axis). **c**, Boxplots of Prosigna ROR values (y-axis) by SSP-ROR (x-axis). **d**, Distributions of Prosigna ROR values and **e**, SSP-ROR values by Prosigna classification for Subtype (four subtypes). **f**, Distributions of Prosigna ROR values and **g**, SSP-ROR values by Prosigna classification for Subtype (four subtypes) for ER+/HER2- breast cancer classified as Luminal A or Luminal B. **h**, Agreement chart and confusion matrix comparing SSP classification (x-axis/columns) with Prosigna classification (y-axis/rows) for ROR risk category and **i**, the two-group ROR stratification according to Bartlett et al. [5]. Boxplot elements: center line (median), bounds of box (upper and lower quartiles), whiskers (1.5x interquartile range).

Supplementary Methods

RNA Sequencing-Based Single Sample Predictors of Molecular Subtype and Risk of Recurrence for Clinical Assessment of Early-Stage Breast Cancer

Johan Staaf[1,*], Jari Häkkinen[1], Cecilia Hegardt[1], Lao H Saal[1], Siker Kimbung[1], Ingrid Hedenfalk[1], Tonje Lien[2,3], Therese Sørlie[2,4], Bjørn Naume[4,5], Hege Russnes[2,3], Rachel Marcone[6,7], Ayyakkannu Ayyanan[6], Cathrin Brisken[6], Rebecka R. Malterling[8], Bengt Asking[8], Helena Olofsson[9, 10], Henrik Lindman[11], Pär-Ola Bendahl[1], Anna Ehinger[1,12], Christer Larsson[13], Niklas Loman[1,14], Lisa Rydén[15,16], Martin Malmberg[14], Åke Borg[1] and Johan Vallon-Christersson[1,*]

[1]Division of Oncology, Department of Clinical Sciences Lund, Lund University, Medicon Village, SE 22381 Lund, Sweden
[2]Department of Cancer Genetics, Institute for Cancer Research, Oslo University Hospital, POB 4953 Nydalen N-0424, Oslo, Norway
[3]Department of Pathology, Oslo University Hospital, POB 4953 Nydalen N-0424, Oslo, Norway
[4]Institute for Clinical Medicine, Faculty of Medicine, University of Oslo, Oslo, Norway
[5]Department of Oncology, Division of Cancer Medicine, Oslo University Hospital, POB 4953 Nydalen N-0424, Oslo, Norway
[6]ISREC-Swiss Institute for Experimental Cancer Research, School of Life Sciences, Ecole Polytechnique Fédérale de Lausanne, CH-1015 Lausanne, Switzerland
[7]Swiss Institute of Bioinformatics, School of Life Sciences, Ecole Polytechnique Fédérale de Lausanne, CH-1005 Lausanne, Switzerland
[8]Department of Surgery, Region Jönköping County, Jönköping, Sweden
[9]Department of Clinical Pathology, Akademiska hospital, Uppsala, Sweden
[10]Department of Pathology, Centre for Clinical Research of Uppsala University, Vastmanland´s Hospital Västerås, Sweden
[11]Department of Immunology, Genetics and Pathology, Uppsala University Hospital, Uppsala, Sweden
[12]Department of Genetics and Pathology, Laboratory Medicine, Region Skåne, Lund, Sweden
[13]Division of Translational Cancer Research, Department of Laboratory Medicine, Lund University, Lund, Sweden
[14]Department of Hematology, Oncology and Radiation Physics, Skåne University Hospital
[15] Division of Surgery, Department of Clinical Sciences, Lund University, Lund, Sweden
[16]Department of Surgery and Gastroenterology, Skåne University Hospital Malmö

Table of content

SCAN-B COHORT 3
Feasibility of RNA sequencing (RNAseq) in a clinical setting with short turnaround time 3
Study-specific cohort stratification 4
GENE EXPRESSION DATA 4
ASSIGNING PAM50 SUBTYPE AND ROR SCORE USING NEAREST CENTROID 5
Nearest centroid (NC) classification and NCN classification. 5
Candidates for selecting NCN reference sets 5
Library protocol correction 5
Normal breast tissue controls 7
Curating the cohort for NCN reference selection 7
TRAINING SSP MODELS USING THE AIMS PROCEDURE 7
Normal and SSP model for suspected low invasive cancer cellularity. 7
Assigning NCN class-labels for intrinsic subtypes to use for SSP training 8
Assigning NCN class-labels for ROR categories to use for SSP training 9
Training SSP models for conventional clinical markers. 10
TIME TO EVENT ANALYSIS 11
EMULATED TREATMENT RECOMMENDATION (ETR) IN PATIENTS WITH ER+/HER2-/N0 AND PT1-2 TUMORS 11
STATISTICS 12
REFERENCES 13
SUPPLEMENTARY METHODS TABLE 1 13

SCAN-B cohort

Feasibility of RNA sequencing (RNAseq) in a clinical setting with short turnaround time

SCAN-B is an observational study with population-based inclusion in that all patients with breast cancer or suspected breast cancer within the catchment area are asked to participate. The SCAN-B material has been accumulated prospectively by continuous patient enrollment and collection of tissue since 2010. Inclusion rates are very high (>90%) over the years and the unbiased nature of inclusion is verified through comparison to the national health care quality assurance registry for breast cancer (NKBC). Whereas collected tissue has been processed with generally very short lead-times from the start of SCAN-B, extracted RNA was initially stored awaiting implementation of the SCAN-B laboratory procedures for RNAseq. When such procedures had been established and tested, the stockpiled RNA was processed and sequenced. Once the backlog of stored RNA was processed the SCAN-B laboratory transitioned to implement real-time continuous processing of collected tissue and RNAseq with short turnaround time. The real-time processing was successfully launched during the fall of 2015 and was in continuous operation until early 2019. Throughout this period, the general turn-around time from tissue arriving at the SCAN-B laboratory to the completion of RNAseq and compiling a result report with gene expression readouts was successfully maintained at less than 1 week (Supplementary methods Figure 1). Since SCAN-B activities were confined to research use only, the turn-around requirements were relaxed during summer and winter holidays, i.e., July/August and December/January (see Supplementary methods Figure 1). Nonetheless, three years of real-time processing by the SCAN-B laboratory demonstrated the feasibility in meeting turnaround

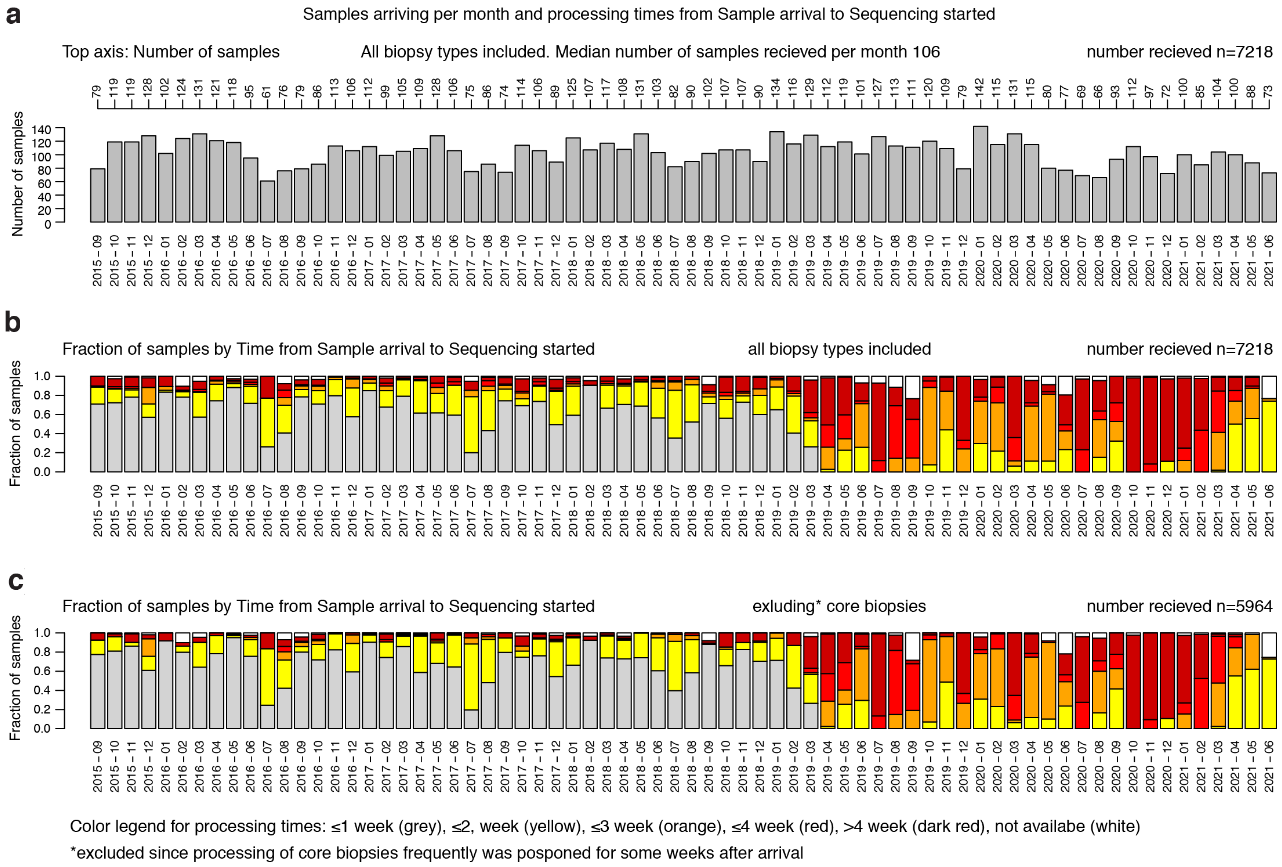

Supplementary methods Figure 1. Number of samples received per month at the SCAN-B laboratory and processing times (September 2015 to June 2021). Real-time processing with short turn-around times was in operation at the SCAN-B laboratory between September 2015 and early 2019. **a**, Number of samples arriving per month including pre-operative core biopsies and biopsied taken from surgery. **b**, Fraction of samples by processing time from arrival to sequencing including both core biopsies and biopsies taken from surgery. **c**, Fraction of samples by processing time from arrival to sequencing for biopsies taken from surgery specimen (excluding core biopsies). In general, the time required from sequencing started to the completion of automated demultiplexing and creation of compiled GEX results reports was <24h. Color coding for processing time: ≤1 week (grey), ≤2, week (yellow), ≤3 week (orange), ≤4 week (red), > 4 week (dark red), not available (white).

time requirements for clinical use regarding tissue sampling and RNAseq. From 2019 the SCAN-B laboratory has continued processing breasts cancer tissue samples but without adhering to short lead-times.

Study-specific cohort stratification

The SCAN-B material used in this study includes a total of 9206 RNAseq derived gene expression profiles (GEXs). The material was divided into separate study cohorts used for training and test/validation. A test cohort suitable for follow-up analysis was selected. The test cohort comprised 2412 patients diagnosed with early-stage surgically resected breast cancer between September 1 2010 and December 31 2013 and with available clinicopathological data for stratification into relevant clinical evaluation groups based on classic clinical markers and treatment. There are no technical replicates included in the test cohort and it is non-redundant, i.e., each included patient is represented by a single GEX. Second, an isolated training cohort was set apart comprising 5857 GEXs from 5711 tissue samples derived from 5250 patients. Most importantly, all training of SSP models was completely confined to the training set and there is no patient overlap between the training cohort and the test cohort. In that respect, the test set is completely independent from the training material and validation can be regarded as independent.

Gene expression data

Gene expression data was derived from RNAseq data from Illumina sequencers using a SCAN-B analysis pipeline based on open source software. Analysis steps were implemented as an automated analysis pipeline in BASE [1] with extension package Reggie (BioRxiv: https://doi.org/10.1101/038976). GRCh38 [2] [grch38], dbSNP [3] [dbsnp], and GENCODE [4] [gencode] are used to create alignment and transcript targets. The analysis steps are listed in sequence (default software parameter values are used except as stated below):

1. Raw base call data is generated with Illumina HiSeq, NextSeq, and NovaSeq sequencers according to manufacturer's instructions (Illumina).
2. Demultiplexing the raw sequencing reads is done with Picard tools [5] ExtractIlluminaBarcodes and IlluminaBasecallsToFastq. Several versions of Picard tools have been used 1.120, 1.128, 2.20.0, 2.20.8, and 2.22.3. Only PF annotated reads are retained during demultiplexing.
3. The demultiplexed sequence files are processed with trimmomatic [6] to remove parts of the readouts that match adapter sequences and to remove poor quality base pair readouts at the end of the reads. Two versions of trimmomatic have been used, versions 0.32 and 0.33. Trimmomatic is run in two passes. First we filter adapters with parameters `ILLUMINACLIP:TruSeq3-PE-2.fa:2:30:12:1:true` and `MINLEN:20`. The second pass is then run with parameters `MAXINFO:40:0.9` and `MINLEN:20`.
4. Reads that match a target of selected genomic sequences are removed. This filtering step remove reads that are considered to be of no use in subsequent analysis – spiked in Enterobacteria phage phiX174 (RefSeq identifier NC_001422.1), ribosomal RNA (RefSeq NR_023363.1, NR_003285.2, NR_003286.2, NR_003287.2, and Genbank X12811.1, U13369.1), repeating sequencing elements (RepeatMasker@UCSC Genome Browser hg38 [2] [genomebrowser]). Bowtie [7] was used as the aligner in this filter step. Several 2.2.x versions of bowtie have been used. Key parameters used are: `--fr -k 1 --local`
5. Alignment is performed by Hisat version 2.1.0 [8]. The GRCh38 human genome assembly is used as the genome target and the transcriptome target is defined by the GENCODE release 27 [4]. The hisat index is augmented using the `--snp` parameter during index creation. dbSNP build 150 is used for all alignments. Key parameters used are: `--fr --no-unal --non-deterministic --novel-splicesite-outfile aligned/splicesites.tsv --rna-strandness RF`
6. Expression estimation is performed with stringtie [9] version 1.3.3b using protein coding transcripts from GENCODE release 27 as transcriptome model. Novel transcripts are discarded. Key parameters used are: `--rf -e`
7. Summary (merge on gene symbol). For collapsing on gene, the sum of expression values of each contributing transcript is calculated.

Assigning PAM50 subtype and ROR score using nearest centroid

Nearest centroid (NC) classification and NCN classification.

PAM50 subtype and ROR score were assigned by nearest centroid (NC) classification using published PAM50 subtype centroids, and largely following the general strategy previously established by Parker et al. [10], i.e., using a reference set of samples for appropriate gene transformation before calculating correlations to PAM50 centroids.

In order to correctly transform gene expression to match the training population from which the PAM50 centroids were originally derived we selected the reference samples by matching the clinicopathological metadata of samples in the original training population defined by Parker et al. to SCAN-B cases. In addition, we extended the general strategy by utilizing multiple reference sets of samples to achieve an NCN classification, i.e., nearest centroid with N reference sets and with N=100. Thus, the reference set selection procedure was repeated 100 times to create a series of 100 static reference sets, each matched to mimic the original training population.

Candidates for selecting NCN reference sets

A subset of the training cohort was chosen as candidates for NCN reference set selection. The candidate cohort was selected to be non-redundant for case (i.e., patient/laterality) and to only include GEX data from samples from invasive breast cancer with available clinicopathological metadata relevant for matching the original PAM50 training population from Parker et al. Included GEX data was also required to meet quality control criteria defined below.

Available clinicopathological metadata requirements:

- Invasive breast cancer
- ER status
- HER2 status
- Lymph node status
- Nottingham grade (NHG) status

RNA and GEX quality control criteria:

- RNA QC (abs) ≥6
- (ALIGNED ≥10M AND fraction duplicates ≤ 0.55) OR UNIQUE READS ≥10M

When multiple GEX assays where available for a case, one assay was randomly selected from the RNA with the highest original concentration by the rational that higher RNA yield would indicate higher tumor cellularity. The resulting cohort of candidates (pam50.reference.pre.cohort) included GEX for a total of 3334 samples distributed between different library protocols as follows: dUTP n=1107, NeoPrep n=938, and TruSeq manual n=1289.

Library protocol correction

GEX data from different library protocols is expected to affect relative gene expression. As centroid classification is relativistic, we constructed a simplistic library protocol adjustment procedure to transform GEX from one protocol-base to another. We first stratified data by library protocol while controlling for clinical subgroups so that clinical subgroup proportions were identical across each stratum. We reasoned that the average gene expression for individual genes should be the same in groups balanced for clinical subgroups when selected from the same overall clinical series. We calculated average gene expression for each gene in respective collection and used the gene specific delta (e.g., see truseq.dutp.diff below) between strata to adjust gene expression from one library protocol to another. Thereby, all gene expression data could be transformed to TruSeq-like expression as a common baseline. We performed SWAMP [11] analysis before and after library protocol correction and confirmed that library protocol signal in the gene expression data was removed, while signal to relevant clinical variables were retained (Supplementary methods Figure 2).

To construct the library protocol adjustment procedure, we used the candidates for NCN reference set selection, i.e., the pam50.reference.pre.cohort. From this data we created three separate cohorts,

each with two groups of library protocols (dUTP/TruSeq, dUTP/NeoPrep, and NeoPrep/TruSeq, respectively) to derive gene specific mean differences between library protocols (Supplementary methods Table 1). Again, the assumption was that by having balanced groups any difference in gene

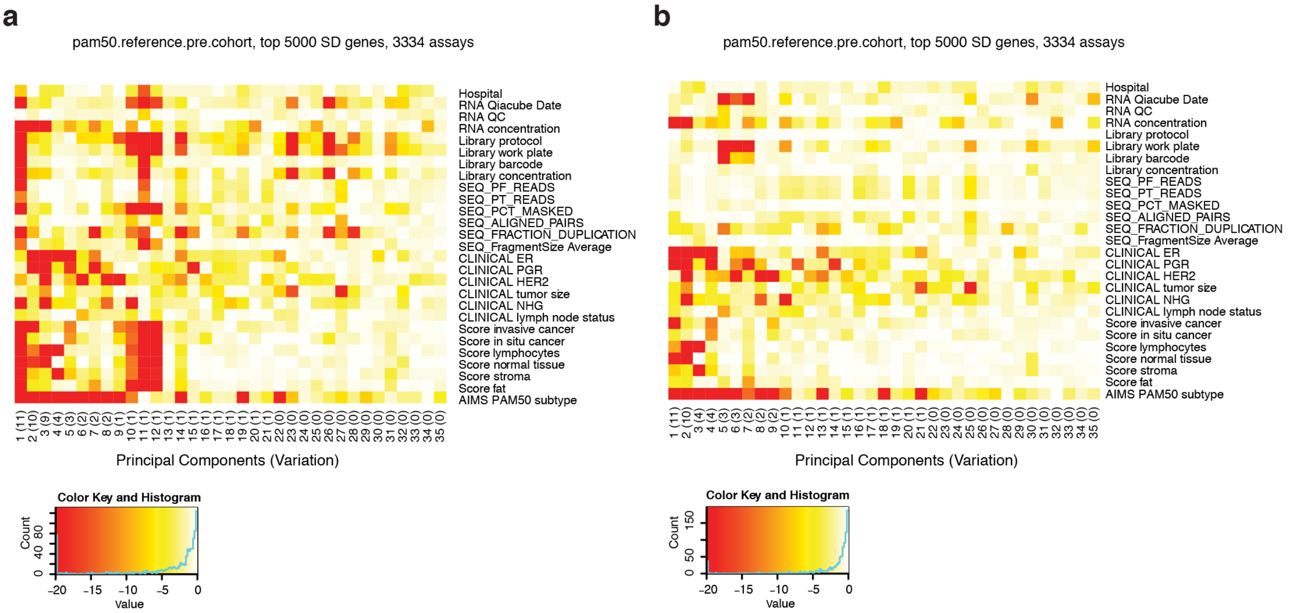

Supplementary methods Figure 2. SWAMP analysis before and after library protocol correction. Analysis is done using the 5000 genes with largest standard deviation measured across the analyzed cohort. **a**, Analysis before library protocol correction. **b**, Analysis after library protocol correction.

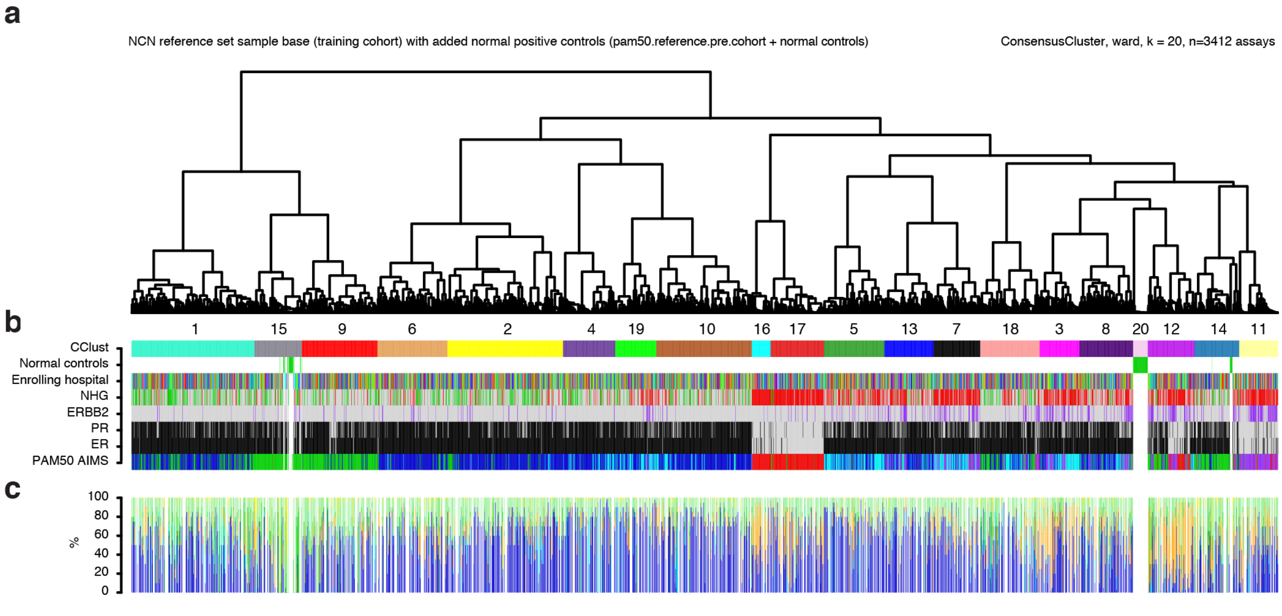

Supplementary methods Figure 3. Consensus clustering of the NCN reference set sample candidate base with the added normal positive GEX controls (pam50.reference.pre.cohort + normal controls) using transposed eigenvectors from PCA analysis of gene expression of all genes (>19000). **a**, Dendrogram from consensus clustering with k=20. Consensus cluster number (1-20) is shown below the dendrogram. **b**, GEX assay annotations. CClust: Consensus cluster. Normal controls: normal tissue control (green). Enrolling hospital: SCAN-B enrollment site. NHG: Grade 1 (green), Grade 2 (grey) Grade 3 (red), not available (white). ERBB2: Positive (purple), Negative (grey), not available (white). PR: Positive (grey), Negative (black), not available (white). ER: Positive (grey), Negative (black), not available (white). AIMS: Subtypes by model from Paquet and Hallett 16, Luminal A (blue), Luminal B (cyan), HER2-enriched (purple), Basal-like (red), Normal-like (green), not available (white). **c**, Bar plot with percentage estimate for cell type from cellularity scoring by a breast cancer pathologist: invasive (dark blue), in-situ (light blue), fat (yellow), immune cells (orange), normal mammary epithelial (dark green), stromal (light green), not available (white). Bars correspond to samples. The scored tissue was taken adjacent to the piece used for RNA-sequencing, as part of SCAN-B laboratory processing, and then formalin-fixed and paraffin-embedded to produce a low-density tissue microarray used for cellularity estimation [17].

expression mean can be attributed to the library protocol. The calculated protocol differences per gene were used as correction factor for converting gene expression between protocols.

For example, protocol difference (per gene) for TruSeq and dUTP, i.e., truseq.dutp.diff, was calculated per gene using:

truseq.dutp.diff = mean(log2(TruSeq+0.1)) - mean(log2(dUTP+0.1))

The idea is that dUTP can be transformed to TruSeq using:

log2(TruSeq+0.1) = log2(dUTP+0.1) + truseq.dutp.diff

The transformation function to transform dUTP GEX to TruSeq(like) GEX per gene:

T = dUTP*2^truseq.dutp.diff + 0.1*(2^truseq.dutp.diff - 1)

To avoid negative values, transformed values were capped at 0, i.e., if T<0 then T was set to 0.

Similar protocol differences (per gene) were also calculated to be able to transform gene expression between TruSeq and NeoPrep and between dUTP and NeoPrep library protocols.

Normal breast tissue controls

Normal breast tissue samples from two sources were included and added to the candidates for NCN reference set selection. Source one included 12 GEX from normal breast tissue specimens collected by pathologists from the extended surgical resection specimen of SCAN-B breast cancer patients. Source two included 66 GEX from non-SCAN-B normal breast tissue specimens obtained from mammoplasty surgery from healthy women.

Curating the cohort for NCN reference selection

In order to evaluate the normal tissue samples and to further critically curate all GEX data before selecting NCN reference sets we performed a consensus clustering using transposed eigenvectors from PCA analysis of GEX including all available genes (Supplementary methods Figure 3). Consensus clustering was performed using Bioconductor package ConsensusClusterPlus [12] evaluating 20 clusters with (1 - Pearson correlation) as distance function and using ward.D2 as hierarchical linkage methods. Subsampling was repeated 800 times sampling 80% of samples (GEX) and 98% of features (genes). A number of the external mammoplasty tissue grouped by themselves in consensus cluster (CC) 20 and were excluded as outliers. A small fraction of the remaining mammoplasty tissue and of the normal tissue from SCAN-B grouped in CC 14 together with other samples of mixed clinicopathological characteristics. The remaining normal samples, of both mammoplasty and SCAN-B origin, were confined to a sub-branch of CC 15 were they intermingled with a number of predominantly ER positive, HER2 negative low-grade SCAN-B samples. All normal samples in consensus cluster 14 and 15 were considered suitable positive normal breast tissue controls, whereas other GEX that grouped in these clusters were deemed as possibly normal-contaminated and excluded from NCN reference set selection. A final curated quality controlled sample base of 3094 tumor tissue and 27 normal controls remained for NCN reference set selections. Data from the curated sample base were used to select 100 static reference sets by matching and bootstrapping. None of the reference sets overlap by more than 30%.

Training SSP models using the AIMS procedure

Normal and SSP model for suspected low invasive cancer cellularity.

Based on the consensus clustering results we reasoned that GEX from tumor samples that grouped in CC 15 were at especially high-risk of being affected by low cancer cellularity in the obtained tissue sample. Thus, it would therefore be beneficial, or at least not detrimental, to exclude those from SSP

training. We therefore first trained an SSP model for CC 15 using *Norm15* and *Rest* as class-labels, i.e., samples that grouped in CC 15 (*Norm15*) versus samples that did not group in that cluster (*Rest*). For training the SSP-CC15 model, a total of 3346 GEX were used (Norm15 n=125 and Rest n=3221). The maximum overall agreement was observed at 27 gene-rules per group by AIMS, although only marginal improvements was seen using >2 rules. The union of unique Entrez ID in selected rules was 54. The overall accuracy in the training set was 96.9%.

To evaluate the SSP-CC15 model in an independent test set we classified a TCGA breast cancer cohort, comprising 994 breast tumors and 106 normal breast tissues analyzed by RNAseq. The SSP-CC15 successfully classified 103 of 106 normal and 980 of 994 tumors for an overall accuracy of 98.5%. We therefore considered the SSP-CC15 an appropriate tool for identifying GEX data potentially affected by low tumor cellularity that could be omitted from further SSP training.
The entire training cohort was classified using the SSP-CC15 with 452 of 5857 (8.4%) GEX classified as *Norm15*, equivalent to data from 446 of 5711 (7.8%) individual tissue samples. Correspondingly, the remaining 5405 GEX were available for further SSP training.

Assigning NCN class-labels for intrinsic subtypes to use for SSP training

The non-TruSeq gene expression data for the NCN static reference sets was transformed to TruSeq-like expression using the gene specific deltas (see section *Library protocol correction* of Supplementary methods). Consequently, before applying the NCN classifier, any non-TruSeq raw gene expression data from individual test samples was first adjusted to TruSeq-like for a common baseline.

Next, when applying the NCN classifier, a test sample is first normalized to a static reference set and then compared to subtype centroids by correlation. The normalization was done per gene by centering, using the mean from the reference set, after first adding 0.1 (to avoid logarithms of 0) to FPKM followed by log2 transformation. Correlation to subtype centroids was done by spearman correlation. Subtype was assigned by nearest centroid (highest correlation), requiring a minimum correlation of 0.2 or otherwise assigned as unclassified. ROR was calculated within the normalized reference set data using centroid correlations, tumor size and proliferation score by the ROR equation (below) as described [13-15], followed by scaling the range to 0-100.

$$ROR_{non\text{-}scaled} = 54.7690*(-0.0067*B + 0.4317*H - 0.3172*LA + 0.4894*LB + 0.1133*T + 0.1981*P + 0.8826)$$

B: correlation to the basal-like centroid
H: correlation to the HER2-enriched centroid
LA: correlation to the Luminal A centroid
LB: correlation to the Luminal B centroid
T: pathologic tumor size coded as 0 (≤20 mm) or 1 (>20 mm)

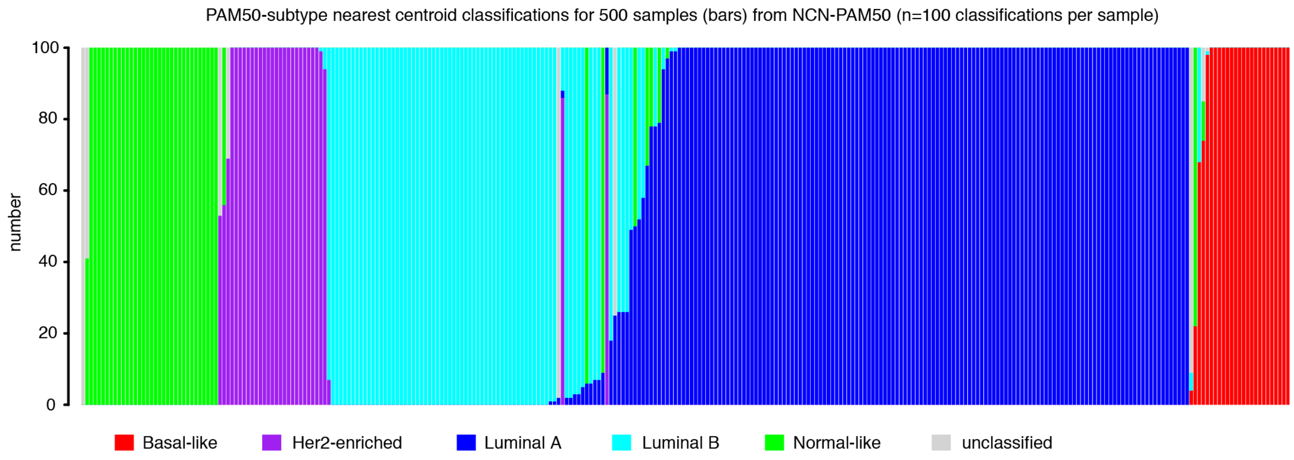

Supplementary methods Figure 4. Individual NCN (n=100) PAM50-subtype classifications for a random sample of 500 GEX assays from the training cohort. Bars correspond to samples.

P: proliferation score (mean expression of an 18-gene proliferation subset from Walden et al. [15] and part of the PAM50 genes)

For each test sample the process is repeated 100 times, using each static reference set for normalization. Scaling of $ROR_{non\text{-}scaled}$ value was done for respective static reference set together with the test sample. Thus, the NCN is essentially 100 separate implementations of a PAM50 classification and accordingly the output for a test sample is a series of 100 individual subtype assignments and scaled ROR scores. For assigning one NCN subtype to a test sample we used the majority vote from the 100 individual classifications and for assigning one NCN ROR score we used the average value of the 100 individual ROR scores. For final SSP training, samples assigned as unclassified by NCN majority vote were excluded.

We applied NCN-PAM50 classification to the entire training cohort to obtain class-labels for training SSP models. We created two sets of class-labels for intrinsic subtype. First, *PAM50-subtype* corresponding to the original intrinsic subtype schema. Second, *Subtype* corresponding to the Prosigna assay with only four categories, omitting the Normal-like centroid when assigning subtype. In most instances, the NCN majority vote assigned subtype with 100% consensus. However, for a number of GEX the vote was not unanimous, but a subtype could still be assigned using a clear majority, and in a small number of instances the vote was close to a draw as exemplified in Supplementary methods Figure 4. These discrepancies demonstrate the relativistic nature of nearest centroid assignments and the risk of classification variance when relying on a single static reference set.

Assigning NCN class-labels for ROR categories to use for SSP training

The ROR score can be related to risk of recurrence using reference patient materials with known clinical outcome. In this manner, predefined cutoffs can be derived for classification into Low, Intermediate, and High risk of recurrence. Different cutoffs are used depending on the nodal status of patients. In addition, the ROR function include the parameter gross tumor size (T) with categorical value 0 or 1 depending on the tumor size from pathologist examination: T=0 for tumors ≤20mm and T=1 for tumors >20mm. Taken together this makes it challenging to construct a categorical SSP model for ROR. To this end, we assigned categorical ROR values to samples through data binning to obtain ROR class-labels that could be used for training SSP models. To determine a suitable and practically useful bin width we related the effect of gross tumor size to the standard deviation (SD) of ROR from our NCN classification. In the training set, respective average SD (SD_{avg}) for ROR, i.e., average of the individual sample SD from the n=100 ROR from NCN, in the two subgroups of gross tumor size was 2.46 for T0 and 2.16 for T1 (Supplementary methods Figure 5 a). We empirically determined the effect of tumor size by calculating ROR twice using alternate settings for parameter T (0 or 1). The SD_{avg} across the cohort for respective T setting was 2.39 for T=0 and 2.29 for T=1

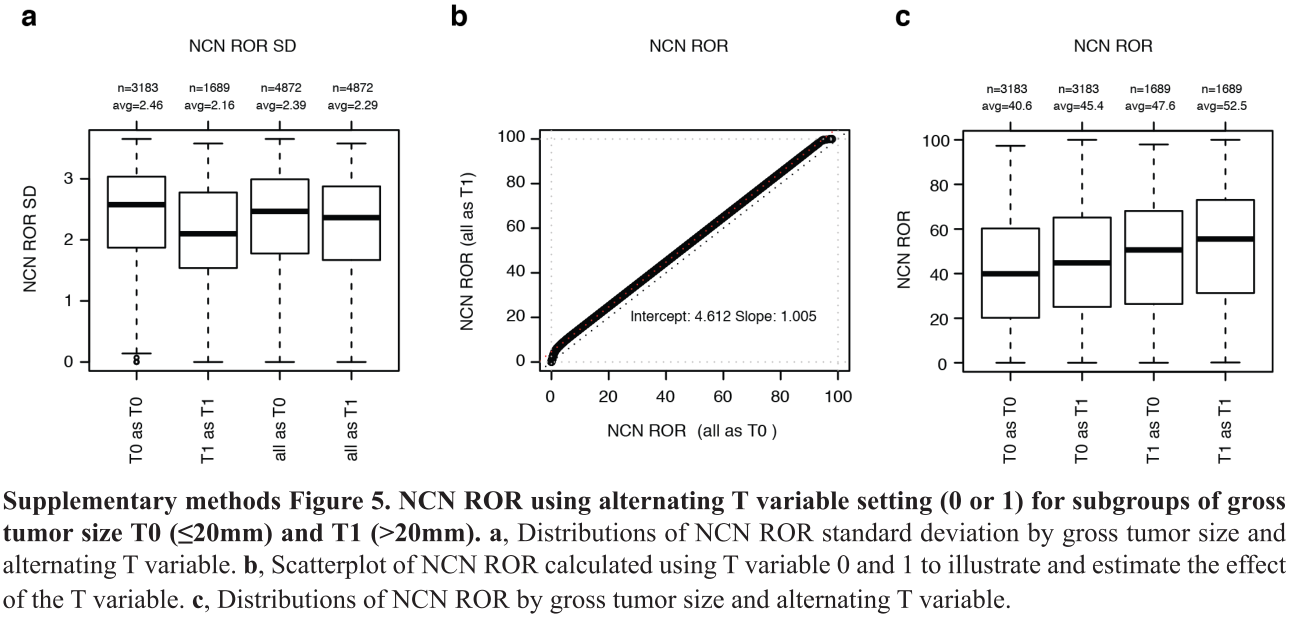

Supplementary methods Figure 5. NCN ROR using alternating T variable setting (0 or 1) for subgroups of gross tumor size T0 (≤20mm) and T1 (>20mm). a, Distributions of NCN ROR standard deviation by gross tumor size and alternating T variable. **b**, Scatterplot of NCN ROR calculated using T variable 0 and 1 to illustrate and estimate the effect of the T variable. **c**, Distributions of NCN ROR by gross tumor size and alternating T variable.

(Supplementary methods Figure 5a). The isolated effect of tumor size was further illustrated by plotting ROR calculated with T=1 versus ROR calculated with T=0 for the same set of samples and was estimated to 4.61 by linear regression (Supplementary methods Figure 5b). This illustrate how alternating the tumor size variable from 0 to 1, i.e., corresponding to tumor size ≤20 mm or >20mm, results in an increase in ROR by 4.61. The estimated effect of tumor size on ROR is within $2SD_{avg}$ across tumors in the training cohort with gross size >20mm (SD_{avg} 2.16 for T1). This could be expressed as that for the SD_{avg} for T1 tumors the ROR adjustment would be within the 95% range of the ROR distribution from the n=100 different static reference sets. The estimated offset was similar for both subgroups of gross tumor size, 4.60 for T0 and 4.64 for T1. The effect of the tumor size variable on ROR was also illustrated by distributions for respective subgroups of gross tumor size using alternate settings for parameter T (0 or 1) (Supplementary methods Figure 5c). One practical implication of the relatively small direct effect of tumor size is that tumor size would not cause samples to shift from *low* to *high* ROR risk category. Instead, only samples close to the cutoffs are at risk of shifting in or out of the *intermediate* category, e.g., from *low* to *intermediate* or vice versa. We therefore used ROR calculated with T=0 for all samples and transformed the numerical value into categorical using a bin width of 5, i.e., ≤5, 6-10, 11-15, .., 91-95, 96-100) to obtain ROR class-labels for training a SSP model. A minimum of 100 cases was required in a bin to include it in training, resulting in a total of 5359 GEX used in training with the following binned ROR labels: <5 n=100, 6-10 n=186, 11-15 n=272, 16-20 n=314, 21-25 n=324, 26-30 n=317, 31-35 n=361, 36-40 n=318, 41-45 n=311, 46-50 n=364, 51-55 n=384, 56-60 n=390, 61-65 n=411, 66-70 n=366, 71-75 n=349, 76-80 n=272, 81-85 n=196, 86-90 n=124). Accordingly, we trained one SSP-ROR model to use for all tumors irrespectively of size ≤20 mm or >20 mm followed by a simple adjustment for gross tumor size variable. Basically, for cases with tumor size ≤20 mm the SSP-ROR assigned score was used as is, whereas for cases with tumor size >20 mm the SSP-ROR assigned score was adjusted with +5 to appropriately account for the effect of the gross tumor size variable.

Training SSP models for conventional clinical markers.

We trained SSP models for the conventional clinical markers: ER, PR, HER2, NHG, and Ki67. Cases in the training cohort with available clinicopathological information were used. Receptor status as determined by respective local pathology department was used. In Sweden, clinical guidelines dictate that tumors with ≤10% of cells with IHC staining for ER and PR status are categorized as negative (Negative). For HER2 status, a case is categorized as negative if IHC staining score <2 or, for patients with IHC 2+ if the ISH status is non-amplified. For training the SSP-ER model, a total of 4786 GEX were used (Negative n=740, and Positive n=4046). The maximum overall agreement was observed at 19 gene-rules per group by AIMS, although only marginal improvements was seen using >3 rules. The union of unique Entrez ID in selected rules was 38. For training the SSP-PR model, a total of 4782 GEX were used (Negative n=1489, and Positive n=3293). The maximum overall agreement was observed already with a single gene-rule that included PR and slowly decreased with increased rules. To retain rule redundancy, we selected to use 3 gene rules. The union of unique Entrez ID in selected rules was 6. For training the SSP-HER2, a total of 4640 GEX were used (Negative n=3986, and Positive n=654). The maximum overall agreement was observed at 8 gene-rules per group. The union of unique Entrez ID in selected rules was 16. For training the SSP-HER2 specific for ER+ cases, a total of 3708 GEX were used (Negative n=3291, and Positive n=417). The maximum overall agreement was observed at 8 gene-rules per group. The union of unique Entrez ID in selected rules was 16. For training the SSP-HER2 specific for ER- cases, a total of 658 GEX were used (Negative n=460, and Positive n=198). The maximum overall agreement was observed at 7 gene-rules per group. The union of unique Entrez ID in selected rules was 14. For training the SSP-Ki67, a total of 3975 GEX were used (High n=2193, and Low n=1782). The maximum overall agreement was observed at 12 gene-rules per group. The union of unique Entrez ID in selected rules was 24. For training the SSP-NHG, a total of 4410 GEX were used (Grade 1 n= 670, Grade 2 n=2191 and Grade 3 n=1549). The maximum overall agreement was observed at 3 gene-rules per group. The union of unique Entrez ID in selected rules was 15.

Time to event analysis

Primary endpoint for time-to-event analysis was distant recurrence-free interval (DRFi). Additional endpoints were overall survival (OS), recurrence-free interval (RFi) and breast cancer-free interval (BCFi). Distant recurrence free interval was defined as the time from breast cancer diagnosis until distant recurrence. Death was regarded as a censoring event. Overall survival was defined as the time from breast cancer diagnosis until death from any cause. For OS, complete follow-up from the Swedish population registry via NKBC was available until 2021. Recurrence free interval was defined as the time from breast cancer diagnosis until any recurrence (local, regional or distant). Death was regarded as a censoring event.

For BCFi included events were: i) any breast cancer recurrence (local, regional or distant), ii) a contralateral breast cancer (invasive or in-situ), and iii) breast cancer related death. For a contralateral breast cancer to be regarded as an event we required that it occurred >90 days from diagnosis. If a case was recorded ≤90 days from diagnosis it was not regarded as a separate event and if it was also classified as invasive the case was excluded from survival analysis. Death other than from breast cancer related death was regarded as a censoring event.

In accordance with the standard operating procedures for reporting to the Swedish National Quality Register (NKBC), recurrence events are reported continuously whereas follow-up for disease-free status is only reported at set time-points after diagnosis (at 1 year and at 5 years after diagnosis). To avoid considerable censoring at 1 year with subsequent overestimation of the recurrence rate, the BCFi follow-up time for recurrence-free patients was extended to the follow-up time for overall-survival. However, to minimize false negative rates for recurrence, a time margin to allow for reporting recurrences to NKBC was applied according to the following:

i) For cases with available extended survival time that were event-free and were the extended survival time was >6 months longer than other available NKBC reported follow-up times we reduce the extended follow-up from survival with 6 months (183 days) to have a safety margin.

ii) For cases that were event-free and were we had extended survival time, but the extended survival time was ≤6 months than available NKBC reported follow-up times, we used the available NKBC reported follow-up time. We also required a minimum of 365 days of available follow-up time for overall survival to assure time for healthcare staff to report events to NKBC.

Extending the follow-up for BCFi as outlined above was assessed as feasible by observed recurrence rates that were in line with national Swedish estimates, and by comparison to a large subset of patients where extended journal review was specifically performed to curate recurrence status. No significant difference in recurrence rate was observed for the journal review group.

Emulated treatment recommendation (ETR) in patients with ER+/HER2-/N0 and pT1-2 tumors

The clinical value of any prognostic stratification is ultimately its influence on disease management such as treatment recommendations. In order to better assess how molecular stratification by, e.g., developed SSP models may affect adjuvant chemotherapy recommendations in a clinical setting we employed a stratification that emulate treatment recommendation (ETR) in patients with ER+/HER2-/N0 and pT1-2 tumors. The ETR schema we used was adapted from available Norwegian guidelines for adjuvant systemic treatment in patients with ER+/HER2-/N0 status when the Prosigna test is available (IS-2945 table 7.4.1 from 08/2020). Our ETR adheres to the actual general recommendations of the Norwegian guidelines but is simplified in that it disregards the guidelines additional considerations for escalation or de-escalation. We employed our ETR to stratify patients into three groups with respect to recommended adjuvant treatment: None, Endo (i.e., endocrine treatment alone for 5-10 years), or ChemoEndo (i.e., adjuvant chemotherapy followed by endocrine treatment for 5-10 years).

The ETR stratification was done as follows:

Tumor size variable pT was assigned using pathology determined tumor size (mm) from NKBC data according to:

Tis = in situ only
T1mi = size ≤1
T1a = size >1 ≤5
T1b = size >5 ≤10
T1c = size >10 ≤20
T2 = size >20 ≤50
T3 = size >50

ER status was categorized using pathology determined ER% staining from NKBC data according to:

ER<50 = ER% staining <50%
ER≥50 = ER% staining ≥50%

Using RNAseq classification for Subtype (LumA, LumB, Her2 or Basal) and ROR risk category (Low, Intermediate, and High) in combination with the pT variable, categorized ER status and pathology determined NHG status from NKBC data we assigned ETR (None, Endo or ChemoEndo) according to:

None = LumA & Low & (T1a OR T1b)
None = LumA & Low & T1c & Grade1
Endo = LumA & Low & T1c & (Grade2 OR Grade3)

Endo = LumA & Low & T2
Endo = LumA & Intermediate & (T1a OR T1b)
Endo = LumA & Intermediate & T1c

Endo = LumA & Intermediate & T2

Endo = (LumB OR Basal OR Her2) & Intermediate & (T1a OR T1b) & ER≥50
ChemoEndo = (LumB OR Basal OR Her2) & Intermediate & (T1a OR T1b) & ER<50

Endo = (LumB OR Basal OR Her2) & Intermediate & T1c & ER≥50
ChemoEndo = (LumB OR Basal OR Her2) & Intermediate & T1c & ER<50

ChemoEndo = (LumB OR Basal OR Her2) & Intermediate & T2 & ER≥50
ChemoEndo = (LumB OR Basal OR Her2) & Intermediate & T2 & ER<50

Endo = High & (T1a OR T1b) & ER≥50
ChemoEndo = High & (T1a OR T1b) & ER<50

ChemoEndo = High & T1c
ChemoEndo = High & T2

All others unassigned.

Statistics

All p-values reported from statistical tests are two-sided if not otherwise specified. Box-plot elements corresponds to: i) center line = median, ii) box limits = upper and lower quartiles, iii) whiskers = 1.5x interquartile range.

References

1. Vallon-Christersson, J., Nordborg, N., Svensson, M. & Hakkinen, J. BASE--2nd generation software for microarray data management and analysis. *BMC Bioinformatics* **10**, 330 (2009).
2. UCSC Genome Browser. http://genome.ucsc.edu/. Accessed:
3. dbSNP. https://www.ncbi.nlm.nih.gov/SNP/. Accessed: 2021. Nov 12
4. Frankish, A.*, et al.* GENCODE reference annotation for the human and mouse genomes. *Nucleic acids research* **47**, D766-D773 (2019).
5. Picard tools. https://broadinstitute.github.io/picard/. Accessed: 2021. Nov 12
6. Bolger, A.M., Lohse, M. & Usadel, B. Trimmomatic: a flexible trimmer for Illumina sequence data. *Bioinformatics* **30**, 2114-2120 (2014).
7. Langmead, B. & Salzberg, S.L. Fast gapped-read alignment with Bowtie 2. *Nature methods* **9**, 357-359 (2012).
8. Kim, D., Paggi, J.M., Park, C., Bennett, C. & Salzberg, S.L. Graph-based genome alignment and genotyping with HISAT2 and HISAT-genotype. *Nature biotechnology* **37**, 907-915 (2019).
9. Pertea, M.*, et al.* StringTie enables improved reconstruction of a transcriptome from RNA-seq reads. *Nature biotechnology* **33**, 290-295 (2015).
10. Parker, J.S.*, et al.* Supervised risk predictor of breast cancer based on intrinsic subtypes. *J Clin Oncol* **27**, 1160-1167 (2009).
11. Lauss, M.*, et al.* Monitoring of technical variation in quantitative high-throughput datasets. *Cancer Inform* **12**, 193-201 (2013).
12. Wilkerson, M.D. & Hayes, D.N. ConsensusClusterPlus: a class discovery tool with confidence assessments and item tracking. *Bioinformatics* **26**, 1572-1573 (2010).
13. Gnant, M.*, et al.* Predicting distant recurrence in receptor-positive breast cancer patients with limited clinicopathological risk: using the PAM50 Risk of Recurrence score in 1478 postmenopausal patients of the ABCSG-8 trial treated with adjuvant endocrine therapy alone. *Ann Oncol* **25**, 339-345 (2014).
14. Filipits, M.*, et al.* The PAM50 risk-of-recurrence score predicts risk for late distant recurrence after endocrine therapy in postmenopausal women with endocrine-responsive early breast cancer. *Clin Cancer Res* **20**, 1298-1305 (2014).
15. Wallden, B.*, et al.* Development and verification of the PAM50-based Prosigna breast cancer gene signature assay. *BMC Med Genomics* **8**, 54 (2015).
16. Paquet, E.R. & Hallett, M.T. Absolute assignment of breast cancer intrinsic molecular subtype. *Journal of the National Cancer Institute* **107**, 357 (2015).
17. Saal, L.H.*, et al.* The Sweden Cancerome Analysis Network - Breast (SCAN-B) Initiative: a large-scale multicenter infrastructure towards implementation of breast cancer genomic analyses in the clinical routine. *Genome Med* **7**, 20 (2015).

Supplementary methods Table 1. GEX cohorts balanced for clinical subgroups for constructing the library adjustment procedure.

COHORT 1
n=1988
dUTP/TruSeq

Clinical subgroup	dUTP	TruSeq
ERpHER2nLNn_Cyto	2	2
ERpHER2nLNn_Endo	346	346
ERpHER2nLNn_EndoCyto	100	100
ERpHER2nLNn_ImmuEndoCyto	2	2
ERpHER2nLNn_NA	1	1
ERpHER2nLNn_None	69	69
ERpHER2nLNp_Endo	130	130
ERpHER2nLNp_EndoCyto	132	132
ERpHER2nLNp_ImmuEndoCyto	2	2
ERpHER2nLNp_None	5	5
HER2pERn_ImmuCyto	31	31
HER2pERn_ImmuEndoCyto	1	1
HER2pERn_NA	2	2
HER2pERn_None	9	9
HER2pERp_Endo	11	11
HER2pERp_EndoCyto	4	4
HER2pERp_ImmuCyto	4	4
HER2pERp_ImmuEndoCyto	56	56
HER2pERp_NA	1	1
HER2pERp_None	3	3
NA_Cyto	1	1
NA_Endo	1	1
TNBC_Cyto	57	57
TNBC_Endo	2	2
TNBC_EndoCyto	1	1
TNBC_NA	2	2
TNBC_None	19	19

COHORT 2
n=1824
dUTP/NeoPrep

Clinical subgroup	dUTP	NeoPrep
ERpHER2nLNn_Cyto	1	1
ERpHER2nLNn_Endo	331	331
ERpHER2nLNn_EndoCyto	100	100
ERpHER2nLNn_ImmuEndoCyto	1	1
ERpHER2nLNn_NA	2	2
ERpHER2nLNn_None	67	67
ERpHER2nLNp_Endo	102	102
ERpHER2nLNp_EndoCyto	132	132
ERpHER2nLNp_None	1	1
HER2pERn_ImmuCyto	21	21
HER2pERn_NA	1	1
HER2pERn_None	3	3
HER2pERp_Endo	9	9
HER2pERp_EndoCyto	1	1
HER2pERp_ImmuEndoCyto	52	52
HER2pERp_NA	1	1
NA_Cyto	1	1
TNBC_Cyto	57	57
TNBC_Endo	1	1
TNBC_EndoCyto	2	2
TNBC_ImmuCyto	1	1
TNBC_NA	2	2
TNBC_None	23	23

COHORT 3
n=1852
NeoPrep/TruSeq

Clinical subgroup	NeoPrep	TruSeq
ERpHER2nLNn_Cyto	1	1
ERpHER2nLNn_Endo	331	331
ERpHER2nLNn_EndoCyto	102	102
ERpHER2nLNn_ImmuEndoCyto	1	1
ERpHER2nLNn_NA	1	1
ERpHER2nLNn_None	67	67
ERpHER2nLNp_Endo	102	102
ERpHER2nLNp_EndoCyto	143	143
ERpHER2nLNp_None	1	1
HER2pERn_ImmuCyto	21	21
HER2pERn_NA	1	1
HER2pERn_None	3	3
HER2pERp_Endo	9	9
HER2pERp_EndoCyto	1	1
HER2pERp_ImmuEndoCyto	52	52
HER2pERp_NA	1	1
NA_Cyto	1	1
TNBC_Cyto	65	65
TNBC_Endo	1	1
TNBC_EndoCyto	1	1
TNBC_NA	2	2
TNBC_None	19	19
